# Anti-inflammatory and antioxidant activity of astragalus polysaccharide in ulcerative colitis: A systematic review and meta-analysis of animal studies

**DOI:** 10.3389/fphar.2022.1043236

**Published:** 2022-12-02

**Authors:** Heng-Chang Hu, Wei Zhang, Pei-Yu Xiong, Li Song, Bo Jia, Xing-Long Liu

**Affiliations:** College of Basic Medicine, Chengdu University of Traditional Chinese Medicine, Chengdu, China

**Keywords:** astragalus polysaccharide, ulcerative colitis, antioxidant, meta-analysis, anti-inflammation, Chinese botanical drug

## Abstract

**Background:** Accumulated evidence indicates that astragalus polysaccharide (APS) may have a beneficial impact on ulcerative colitis (UC) by suppressing inflammation and decreasing oxidative stress. Nevertheless, the credibility of the evidence for this practice is unclear. Therefore, we intended to conduct a systematic review and meta-analysis of animal studies to assess the anti-inflammatory and antioxidant activity of APS when used in the treatment of UC.

**Methods:** Electronic bibliographic databases including PubMed, EMBASE, Web of Science, Chinese Biomedical Literature (CBM), Wanfang Database, CQVIP Database and China National Knowledge Infrastructure (CNKI) were retrieved for relevant animal studies. The methodological quality of animal studies was evaluated based on the SYstematic Review Center for Laboratory animal Experimentation (SYRCLE’s RoB tool). A meta-analysis was performed according to the Cochrane Handbook for Systematic Reviews of Interventions by using STATA 12.0 software. This study was registered with PROSPERO, number CRD42021272595.

**Results:** Twenty qualified publications involving 591 animals were included in this study. There was a significant association of APS with levels of disease activity index (DAI), colon macroscopic damage index (CMDI), colon histopathologic score (CHS), myeloperoxidase (MPO), tumor necrosis factor-α (TNF-α), interleukin-6 (IL-6), interleukin-1β (IL-1β), superoxide dismutase (SOD) and malondialdehyde (MDA) compared with that in the control group. Sensitivity analysis that eliminated one study at each stage did not change these results. Egger’s test and funnel plot showed that publication bias was existed.

**Conclusion:** In this meta-analysis, APS treatment significantly mitigated colonic damage by reducing the levels of MPO, TNF-α, IL-6, IL-1β, and MDA and recovering the SOD activity. These results demonstrated a protective role of APS in the treatment of UC and showed that the anti-inflammatory and antioxidant activity were implicated in the underlying mechanisms. Hence, APS may represent a promising candidate for treating UC. However, due to potential publication bias, a cautious interpretation is needed.

**Systematic Review Registration:** (https://www.crd.york.ac.uk/PROSPERO/).

## Introduction

Ulcerative colitis (UC) is an idiopathic, chronic inflammatory bowel disease affecting rectum and colon (Kobayashi et al., 2020), and it is characterized by remitting and relapsing mucosal inflammation starting in the rectum and extending proximally in the colon in a continuous way (Ungaro et al., 2017). Multiple factors, such as mucosal immune dysregulation, genetic predisposition and environmental factors, have been suggested to contribute to UC pathogenesis (Danese and Fiocchi, 2011; Jostins et al., 2012; Liu et al., 2015; Bernstein et al., 2016). The incidence of UC ranges from 1.2 to 20.3 cases per 100,000 persons per year, and its prevalence is 7.6–246.0 cases per 100,000 persons per year (Loftus, 2004). Because of higher incidence and prevalence, the burden of UC on society and families will continue to grow worldwide. Current treatments for UC patients rely on the 5-aminosalicylates, corticosteroids, thiopurines (azathioprine or 6-mercaptopurine), biological drugs (anti-TNF-α or anti-integrin agents) and surgery (Kornbluth and Sachar, 2010; Bressler et al., 2015; Harbord et al., 2017). However, these strategies are insufficient to achieve better effectiveness in a substantial proportion of patients and often lead to adverse effects. Anti-TNF-α agents could increase the risk of pulmonary tuberculosis and non-melanoma skin cancer (Duan et al., 2021). Thiopurines are associated with liver toxicity, increased risk of non-melanoma skin cancer and lymphoma (Harbord et al., 2017). Tofacitinib (JAK inhibitors) could increase the risk of severe infection and malignant tumor (Olivera et al., 2017). Moreover, patients remain reluctant to accept surgical management due to complicated complications and considerably high cost. Given the limitations and disadvantages of current UC therapy, it is imperative to establish the novel treatment approaches for the management of UC.

As one of the primary contemporary alternative medicines, Chinese botanical drug (CBD) and its active components as a promising candidate become the focus of UC drug research. In contrast to Western medicine, CBD and its active components have been shown to target multiple pathogenic mechanisms that are crucially involved in the occurrence and development of UC. In addition, CBD and its active components provide the advantages of less adverse reactions, wide availability, low cost and good tolerance. Therefore, identifying CBD and its active components exhibiting colonic protective effects may be the important direction of drug research.

Astragalus polysaccharide (APS) is the major bioactive ingredient isolated from the CBD *Astragalus mongholicus* Bunge (Fabaceae; *Astragalus mongholicus* radix), has been traditionally used for the treatment of hypoimmunity, chronic fatigue and tumour (Shan and Ye, 2007; Yan et al., 2017; Wang et al., 2021). Currently, an increasing number of preclinical animal studies focus on the therapeutic effects and mechanisms of APS on experimental UC. Several animal studies indicated that APS treatment could alleviate UC by reducing MDA and increasing SOD levels in UC mice models (Song et al., 2021a; Tang et al., 2021). In addition, APS was suggested to relieve colon damage *via* regulating MPO activity in the rats with TNBS-induced UC (Zang et al., 2017; Zang et al., 2018). Furthermore, APS also could attenuate the TNBS- mediated UC symptoms in animals through a decrease in the inflammatory cytokines, including TNF-α, IL-1β and IL-6 (Li and Gong, 2016; Zang et al., 2017). Results of these animal experiments revealed a beneficial therapeutic effect of APS in the treatment of UC and indicated that the anti-inflammatory and antioxidant activity were implicated in the pivotal mechanism. However, these findings of individual animal experiment are often influenced by various factors, such as dosage, intervention duration, small sample sizes and different methods of APS preparation, thus it is insufficient to draw definitive conclusions about the anti-inflammatory or antioxidant properties of APS in the management of UC based on this poor evidence. In addition, time-response effects and dose-response effects play an important role in the treatment of UC, but it is hard to determine the appropriate intervention duration and dosage of APS based on individual animal study. Furthermore, methodological quality and publication bias in animal studies are still unclear, which may exaggerate the therapeutic effects of APS. These problems should be clarified in order to enhance the therapeutic effects of APS and lower drug-induced risks.

Therefore, we conducted a systematic review and meta-analysis of preclinical animal studies to assess APS for the treatment of UC. The purposes of this study were to 1) provide reasonable evidence to confirm the anti-inflammatory and antioxidant activity of APS in the treatment of UC, 2) explore the appropriate intervention duration and dosage of APS on UC, and 3) provide an assessment of methodological quality and publication bias of the animal studies.

## Methods

This systematic review and meta-analysis was performed according to the Cochrane Handbook for Systematic Reviews of Interventions and reported based on Preferred Reporting Items for Systematic Reviews and Meta-analyses guidelines. The protocol for this meta-analysis was registered with the PROSPERO (CRD42021272595).

### Search strategies

Electronic bibliographic databases including PubMed, EMBASE, Web of Science, CBM, Wanfang Database, CQVIP Database and CNKI were searched for relevant animal studies published from January 2000 to December 2021. Furthermore, the language was limited to Chinese and English. Medical subject headings (MeSH) with free words were employed in English databases. The relevant terms were as follows: Participants (Colitis, Ulcerative [MeSH], Ulcerative colitis, Ulcer colonitis, Colitis gravis, UC; Intervention (Astragalus Polysacharin, Astragalus polysaccharide, APS). Moreover, Chinese database were searched using the aforementioned search terms in Chinese.

### Inclusion criteria

1) Participants: all animal models with UC; 2) Intervention: all dosage and duration of APS are eligible for inclusion; 3) Control group: same solvent, no intervention, etc.; 4) Outcomes: DAI, CMDI and CHS were the primary outcomes, TNF-α, IL-6, IL-1β, MPO, SOD, and MDA were the secondary outcome measures; 5) Study design: randomized controlled studies; 6) Language: Chinese and English.

### Exclusion criteria

1) Participants: clinical trials, *in vitro* studies, etc.; 2) Control group: other Chinese botanical drug; 3) Study design: case report, case-control studies, studies without a separate control group and cross-over studies; 4) Not an original full research paper (e.g., conference proceedings, review, abstracts); 5) Animal studies without full text; 6) Duplicate publication.

### Study selection and data extraction

Screening were conducted in two phases. In the first phase, title and abstract of study were screened independently by two reviewers to identify study that potentially meet the inclusion criteria outlined above. In the second phase, full text of these potentially eligible studies were independently assessed for eligibility by two reviewer authors. Disagreements about whether a controversial study should be included were resolved with a third reviewer through discussion.

Two reviewers extracted the following data independently from included studies: 1) Basic characteristics: first author’s surname and year of publication; 2) Information on participants: species, sample size, weight and UC models in the experimental group and control group; 3) Information on APS treatment: dosage and intervention duration; 4) Outcome measures: DAI, CMDI, CHS, SOD, MDA, MPO, TNF-α, IL-6, and IL-1β. All the outcome measures were continuous variable, so the mean and the standard deviation for experimental group and control group were extracted. For study with multiple experimental groups sharing one control group, then this control group was divided up approximately evenly among the comparisons and each pair-wise comparison was entered into the meta-analysis (Higgins et al., 2019). In case outcome measures were presented at multiple time points, then the data was extracted from the last time point. Any controversy between reviewer authors over the data extraction was resolved with a third reviewer through discussion.

### Quality assessment

The methodological quality of included animal studies was evaluated on the basis of the SYRCLE’s RoB tool. The SYRCLE’s RoB tool for animal experiments involves 10 entries based on six types of bias: 1) Sequence generation (selection bias); 2) Baseline characteristics (selection bias); 3) Allocation concealment (selection bias); 4) Random housing (performance bias); 5) Blinding (performance bias); 6) Random outcome assessment (detection bias); 7) Blinding (detection bias); 8) Incomplete outcome data (attrition bias); 9) Selective outcome reporting (reporting bias); 10) Other sources of bias (other). The results of the evaluation are “yes,” “no” and “unclear,” representing “low risk of bias,” “high risk of bias” and “insufficient details have been reported to assess the risk of bias properly” (Hooijmans et al., 2014).

Two reviewers conducted methodological quality evaluation independently. Moreover, disagreement between reviewers over the quality assessment was resolved with a third reviewer through discussion.

### Statistical analysis

All the outcome measures were continuous variable (e.g., DAI, CMDI, and CHS), so standardized mean difference (SMD) was considered to describe the effect sizes of the intervention effect. Random-effects (DerSimonian and Laird) method was employed for this meta-analysis because this model incorporated between-study variability and provided more conservative pooled estimates (Becic and Studenik, 2018). To identify and measure between-study heterogeneity, the chi-squared test and *I* ^*2*^ statistics (*I* ^*2*^ describes the percentage of the variability in effect estimates) were implemented. The chi-squared test with a significance level of *α* = 0.1 was used as statistical measure of heterogeneity. 0% < *I* ^*2*^ < 40%: might not be important; 30% < *I* ^*2*^ < 60%: may represent moderate heterogeneity; 50% < *I* ^*2*^ < 90%: may represent substantial heterogeneity; 75% < *I* ^*2*^ < 100%: considerable heterogeneity (Higgins et al., 2019). Subgroup analysis was performed to explore the potential sources of heterogeneity and the influence of several factors on the pooled effect sizes based on following variables if there were adequate studies: intervention duration (≤ 7 days, > 7 days), dosage (low ≤ 100 mg/kg, 100 < medium ≤ 200 mg/kg, high > 200 mg/kg), species (rat, mice), UC models (DSS, DNCB, and TNBS). Sensitivity analysis was performed to assess the robustness of the pooled results by removing a single study at each stage. Publication bias was assessed using the funnel plot as well as the Egger’s test (Egger et al., 1997) if there were at least 10 studies for each outcome. With regard to Egger’s test, a *p*-value < 0.05 was considered as statistically significant (Egger et al., 1997). Meta-analysis, subgroup analysis and sensitivity analysis were performed by using STATA 12.0 software.

## Results

### Study inclusion

**FIGURE 1:**
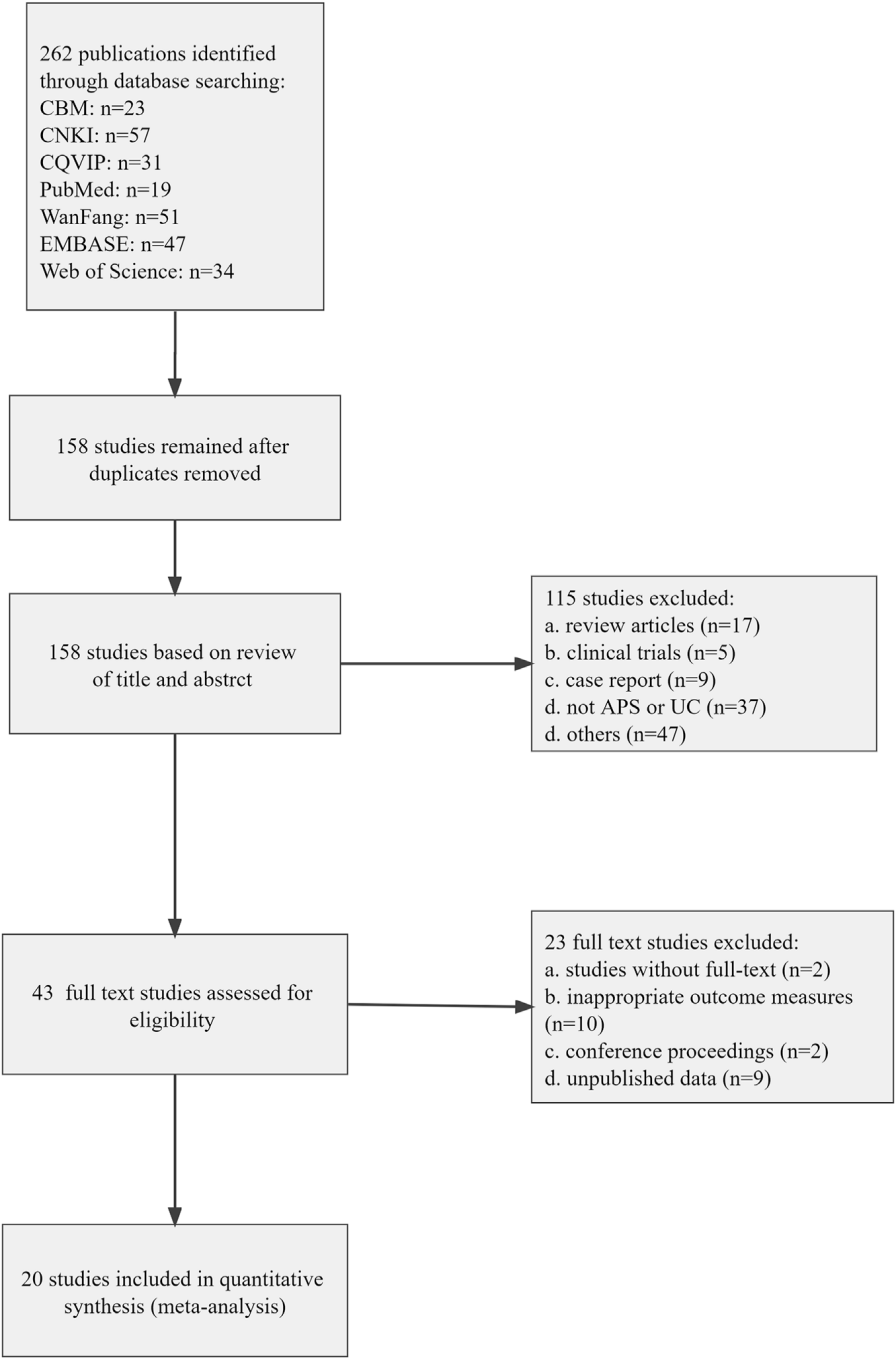
Flow diagram of the study selection process for this review.

A total number of 262 animal studies were identified based on the databases searching for systematic review and meta-analysis. After removing duplicates, 158 publications remained. While screening titles and abstracts, 115 animal studies were eliminated due to the following reasons: 1) review articles; 2) clinical trials; 3) case report; 4) not APS or UC; 5) others (e.g., research on the design, preparation and evaluation of pharmaceutical preparations and extraction of active ingredients of the drug). Then, the full-text screening of the 43 remaining publications revealed that 23 studies were unqualified because of the following reasons: 1) studies without full text (*n* = 2); 2) inappropriate outcome measures (*n* = 10); 3) conference proceedings (*n* = 2); 4) unpublished data (*n* = 9). Ultimately, 20 eligible publications were incorporated in the systematic review and meta-analysis. The process of study selection was provided in Figure 1.

### Study characteristics

Twenty eligible studies including 39 pair-wise comparisons were published between 2010 and 2021. A total of 591 animals were included in these studies. All animals in the experimental group was 393 and that in the control group was 198. Animal species including rat and mice were included in meta-analysis, six studies (30%) of all which used mice, and 14 studies (70%) used rats. The weight of rats ranged from 130 to 250 g in all studies and that of mice ranged from 18 to 22 g, two studies did not report the weight of animals. There were three types of animal models in these studies, namely TNBS-induced UC (65%), DNCB-induced UC (10%), and DSS-mediated UC (25%).

Three levels (low, middle, and high) of APS dosage were implemented in these studies and the dosage ranged from 100 to 2000 mg/kg, two studies did not report APS dose. Control group mainly consisted of water, saline and no intervention. Ten studies (50%) selected saline as control intervention, five studies (25%) adopted water, and the rest five studies (25%) had no intervention. The intervention duration included short duration (≤ 7 days) and long duration (> 7 days). Twelve studies (60%) applied long duration and eight studies (40%) utilized short duration. The characteristics of 20 eligible studies were displayed in Table 1 (Gao et al., 2010; Dai et al., 2011; Guo et al., 2013; Yang et al., 2014; Zhao et al., 2015; Li and Gong, 2016; Zhao et al., 2016; Lv et al., 2017; Zang et al., 2017; Liu, 2018; Zang et al., 2018; Zhao et al., 2018; Chen and Zhu, 2019; Pan et al., 2019; Shen et al., 2019; Yan et al., 2020; Song et al., 2021a; Song et al., 2021b; Tang et al., 2021; Zhong et al., 2021).

**TABLE 1:** Characteristics of the included studies.

Study year	*n* = (T, C)	Species	Weight (g)	UC models	APS dose (mg/kg/d)	Duration (day)	Control group	Outcome index
Gao et al. (2010)	8/8, 8	Rat	200–250	TNBS	500/1,000	14	Saline	1. DAI 2. CMDI 3. CHS. 4. MPO
Dai et al. (2011)	8, 8	Rat	180–220	TNBS	750	10	No intervention	1. DAI 2. CMDI 3. CHS. 4. MPO
Guo et al. (2013)	9/10/10, 9	Rat	130–170	DNCB	2000/1000/500	14	Water	1. DAI 2. CMDI
Yang et al. (2014)	9/9, 9	Rat	180–220	TNBS	100/200	7	Saline	1. DAI 2. CMDI 3. CHS. 4. TNF-α 5. IL-1β
Zhao et al. (2015)	10, 10	Rat	200–220	TNBS	400	7	Saline	1. CMDI 2. CHS
Zhao et al. (2016)	8, 8	Rat	200–220	TNBS	400	7	Saline	1. CMDI 2. CHS 3. IL-6
Li and Gong (2016)	12/12/12, 12	Rat	198–208	TNBS	100/200/400	14	Saline	1. DAI 2. CMDI 3. SOD. 4. MDA 5. MPO 6. IL-6. 7. TNF-α
Lv et al. (2017)	10, 10	Mice	NR	DSS	200	3	Saline	1. DAI 2. CHS 3. MPO. 4. IL-1β 5. TNF-α 6. IL-6
Zang et al. (2017)	8/8/8, 8	Rat	180–200	TNBS	100/200/400	10	No intervention	1. DAI 2. CHS 3. MPO. 4. IL-1β 5. TNF-α
Liu (2018)	10/10/10, 10	Rat	180–200	TNBS	100/200/400	10	No intervention	1. DAI 2. MPO. 3. IL-1β 4. TNF-α
Zang et al. (2018)	10/10/10, 10	Rat	180–220	TNBS	100/200/400	7	Saline	1. CHS 2. MPO 3.TNF-α
Zhao et al. (2018)	10, 10	Mice	18–22	TNBS	200	7	No intervention	1. CHS
Shen et al. (2019)	10/10, 10	Rat	180–220	TNBS	100/400	10	No intervention	1. DAI 2. CMDI 3. MPO. 4. IL-1β 5. TNF-α
Chen and Zhu, (2019)	8, 7	Rat	130–170	DNCB	2000	14	Water	1. DAI 2. CMDI
Pan et al. (2019)	18, 18	Rat	180–200	TNBS	200	10	Saline	1. DAI 2. CMDI 3. CHS
Yan et al. (2020)	13, 10	Rat	190–210	TNBS	NR	28	Water	1. DAI 2. CMDI 3. MPO. 4. SOD 5. MDA 6. IL-1β. 7. TNF-α
Song et al. (2021a)	15/15/15, 15	Mice	20–22	DSS	100/200/400	14	Saline	1. DAI 2. CHS 3. MPO. 4. SOD 5. MDA. 6. TNF-α 7. IL-6
Song et al. (2021b)	8/8/8, 8	Mice	18–22	DSS	100/200/400	10	Water	1. DAI
Tang et al. (2021)	8/8, 8	Mice	NR	DSS	300/600	7	Saline	1. DAI 2. SOD 3. TNF-α. 4. IL-1β 5. MDA 6. IL-6
Zhong et al. (2021)	10/10, 10	Mice	18.5–21.5	DSS	NR	7	Water	1. DAI 2. SOD 3. MDA. 4. MPO 5. IL-1β 6. IL-6. 7. TNF-α

Abbreviations: DNCB, dinitro-chlorobenzene; DSS, dextran sodium sulfate; NR, no report; TNBS, 2, 4, 6-trinitrobenzene sulfonic acid.

In this review, 15 original studies selected APS supplied by pharmaceutical company, four original studies prepared APS by themselves (Lv et al., 2017; Liu, 2018; Tang et al., 2021; Zhong et al., 2021), and one study did not report the source of APS. In the four original studies mentioned above, the preparation process of APS was as follows: 1) Lv et al., 2017: ① extraction with distilled water; ② deproteinization with sevage reagent; ③ precipitation with three times the volume of 95% ethanol; ④ polysaccharides precipitation was gathered by centrifugation; ⑤ wash with 95% ethanol and anhydrou -s ethanol; ⑥ suction and lyophilization. 2) Liu, 2018: ① extraction with distilled water; ② centrifugation; ③ deproteinization with sevage reagent; ④ precipitation with anhydrous ethanol; ⑤ wash with anhydrous ethanol, acetone and anhydrous ether; ⑥ vacuum drying. 3) Tang et al., 2021: ① extraction with distilled water; ② centrifugation; ③ precipitation with anhydrous ethanol; ④: centrifugation; ⑤ wash with anhydrous ethanol, acetone and anhydrous ether; ⑥ vacuum drying. 4) Zhong et al., 2021: this original study did not explain the specific preparation process, and only mentioned the application of ultrasound-assisted extraction and water extraction. A summary table describing the APS was outlined in Table 2.

**TABLE 2:** A summary table describing the APS.

Study year	Compound, concentration	Source and batch number	Purity	Quality control reported?
Gao et al. (2010)	Pure compound	Shaanxi Scipha Biotechnology Co., Ltd., Shaanxi, China (HQ090430)	90%	NR
Dai et al. (2011)	Pure compound	Shaanxi Scipha Biotechnology Co., Ltd., Shaanxi, China (HQ090430)	90%	NR
Guo et al. (2013)	Pure compound	Nanjing ZeLang Medical Technology Co., Ltd., Nanjing, China (20,110,128)	90%	NR
Yang et al. (2014)	NR	NR	NR	NR
Zhao et al. (2015)	Pure compound	Shaanxi Scipha Biotechnology Co., Ltd., Shaanxi, China (HQ090312)	>98%	Y- HPLC
Zhao et al. (2016)	Pure compound	Shaanxi Scipha Biotechnology Co., Ltd., Shaanxi, China (HQ090312)	>98%	Y- HPLC
Li and Gong (2016)	Pure compound	Shaanxi Scipha Biotechnology Co., Ltd., Shaanxi, China (HQ090312)	>98%	Y- HPLC
Lv et al. (2017)	Pure compound	Purified by Lv et al. (2017)	87.4%	Y- HPLC
Zang et al. (2017)	Pure compound	Dalian Meilun Biotechnology Co., Ltd., Liaoning, China (A0418A)	68%	NR
Liu (2018)	Pure compound	Purified by Liu. (2018)	NR	NR
Zang et al. (2018)	Pure compound	Dalian Meilun Biotechnology Co., Ltd., Liaoning, China (A0418A)	70%	NR
Zhao et al. (2018)	Pure compound	Shaanxi Scipha Biotechnology Co., Ltd., Shaanxi, China	NR	NR
Shen et al. (2019)	Pure compound	Dalian Meilun Biotechnology Co., Ltd., Liaoning, China (A0418A)	68%	NR
Chen and Zhu (2019)	Pure compound	Hangzhou Hoops Biotechnology Co., Ltd., Zhejiang, China	90%	NR
Pan et al. (2019)	Pure compound	Dalian Meilun Biotechnology Co., Ltd., Liaoning, China	50%	NR
Yan et al. (2020)	Pure compound	Fuzhou Rimian Technology Development Co., Ltd., Fujian, China	NR	NR
Song et al. (2021a)	Pure compound	Nanjing ZeLang Medical Technology Co., Ltd., Nanjing, China	>90%	NR
Song et al. (2021b)	Pure compound	Shanghai Yuanye Biotechnology Co., Ltd., Shanghai, China	≥98%	NR
Tang et al. (2021)	Pure compound	Purified by Tang et al. (2021)	NR	NR
Zhong et al. (2021)	Pure compound	Purified by Zhong et al. (2021)	96.82%	NR

Abbreviations: HPLC, high performance liquid chromatography; NR, no report.

### Study quality

Random allocation to the experimental group and control group was clarified in 17 studies (88%), and the remaining three studies did not mention the methods of allocation. None of the studies mentioned the distribution of baseline characteristics of animals between the experimental group and control group. None of the studies reported the application of allocation concealment. Random housing, blinding (performance bias) and random outcome assessment were not clarified in all studies. No study described blinding (detection bias). All these studies had complete outcome data and reported expected outcomes. With regard to other sources of bias, four studies (20%) stated that there was no conflict of interest among the authors, the rest 16 studies (80%) did not report it. The methodological quality of included studies was displayed in Table 3.

**TABLE 3:** Risk of bias of included studies.

Study year	(1)	(2)	(3)	(4)	(5)	(6)	(7)	(8)	(9)	(10)
Gao et al. (2010)	Y	U	U	U	U	U	N	Y	Y	U
Dai et al. (2011)	Y	U	U	U	U	U	N	Y	Y	U
Guo et al. (2013)	Y	U	U	U	U	U	N	Y	Y	U
Yang et al. (2014)	Y	U	U	U	U	U	N	Y	Y	U
Zhao et al. (2015)	Y	U	U	U	U	U	N	Y	Y	U
Zhao et al. (2016)	Y	U	U	U	U	U	N	Y	Y	Y
Li and Gong (2016)	Y	U	U	U	U	U	N	Y	Y	U
Lv et al. (2017)	Y	U	U	U	U	U	N	Y	Y	Y
Zang et al. (2017)	U	U	U	U	U	U	N	Y	Y	U
Liu (2018)	U	U	U	U	U	U	N	Y	Y	U
Zang et al. (2018)	Y	U	U	U	U	U	N	Y	Y	U
Zhao et al. (2018)	Y	U	U	U	U	U	N	Y	Y	U
Shen et al. (2019)	Y	U	U	U	U	U	N	Y	Y	U
Chen and Zhu (2019)	Y	U	U	U	U	U	N	Y	Y	U
Pan et al. (2019)	Y	U	U	U	U	U	N	Y	Y	U
Yan et al. (2020)	Y	U	U	U	U	U	N	Y	Y	Y
Song et al. (2021a)	Y	U	U	U	U	U	N	Y	Y	U
Song et al. (2021b)	Y	U	U	U	U	U	N	Y	Y	U
Tang et al. (2021)	U	U	U	U	U	U	N	Y	Y	Y
Zhong et al. (2021)	Y	U	U	U	U	U	N	Y	Y	U

1) sequence generation; 2) baseline characteristics; 3) allocation concealment; 4) random housing; 5) blinding (performance bias); 6) random outcome assessment; 7) blinding (detection bias); 8) incomplete outcome data; 9) selective outcome reporting; 10) other sources of bias; Y, yes; N, no; U, unclear.

### Effectiveness

**FIGURE 2:**
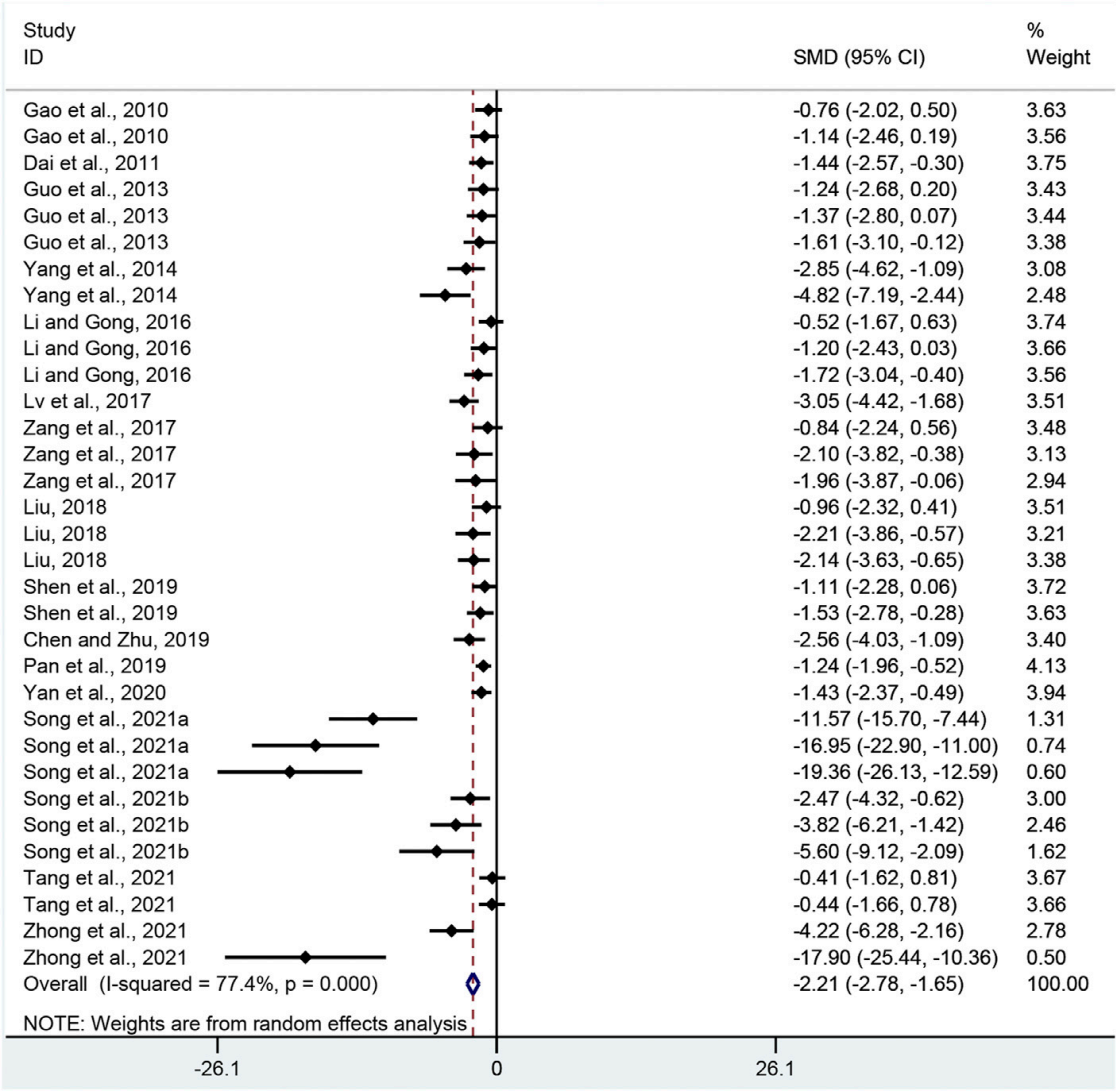
Pooled estimate of DAI with APS.

DAI: Thirty-three pair-wise comparisons reported the influence of APS on DAI. The pooled results showed that APS could significantly decrease DAI scores compared with the control group (SMD = −2.21, 95% CI [−2.78, −1.65], *p* = 0.000; Heterogeneity: Chi^2^ = 141.49, *p* = 0.000; *I* ^2^ = 77.4% Figure 2).

**FIGURE 3:**
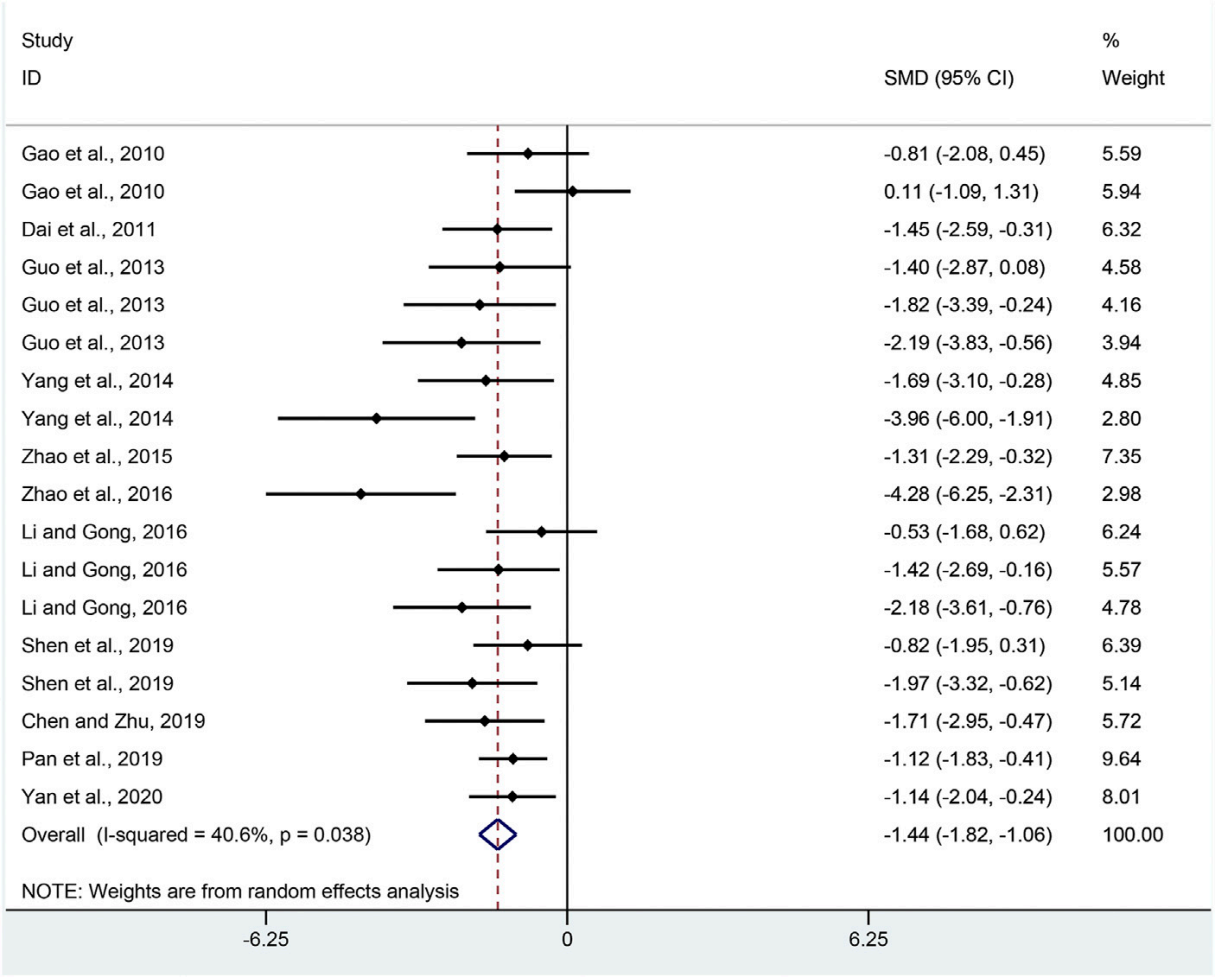
Pooled estimate of CMDI with APS.

CMDI: Combining effect sizes from 18 pair-wise comparisons, a significant decrease in CMDI scores was observed after APS treatment, compared to that in the control group (SMD = −1.44, 95% CI [−1.82, −1.06], *p* = 0.000; Heterogeneity: Chi^2^ = 28.61, *p* = 0.038; *I* ^2^ = 40.6% Figure 3).

**FIGURE 4:**
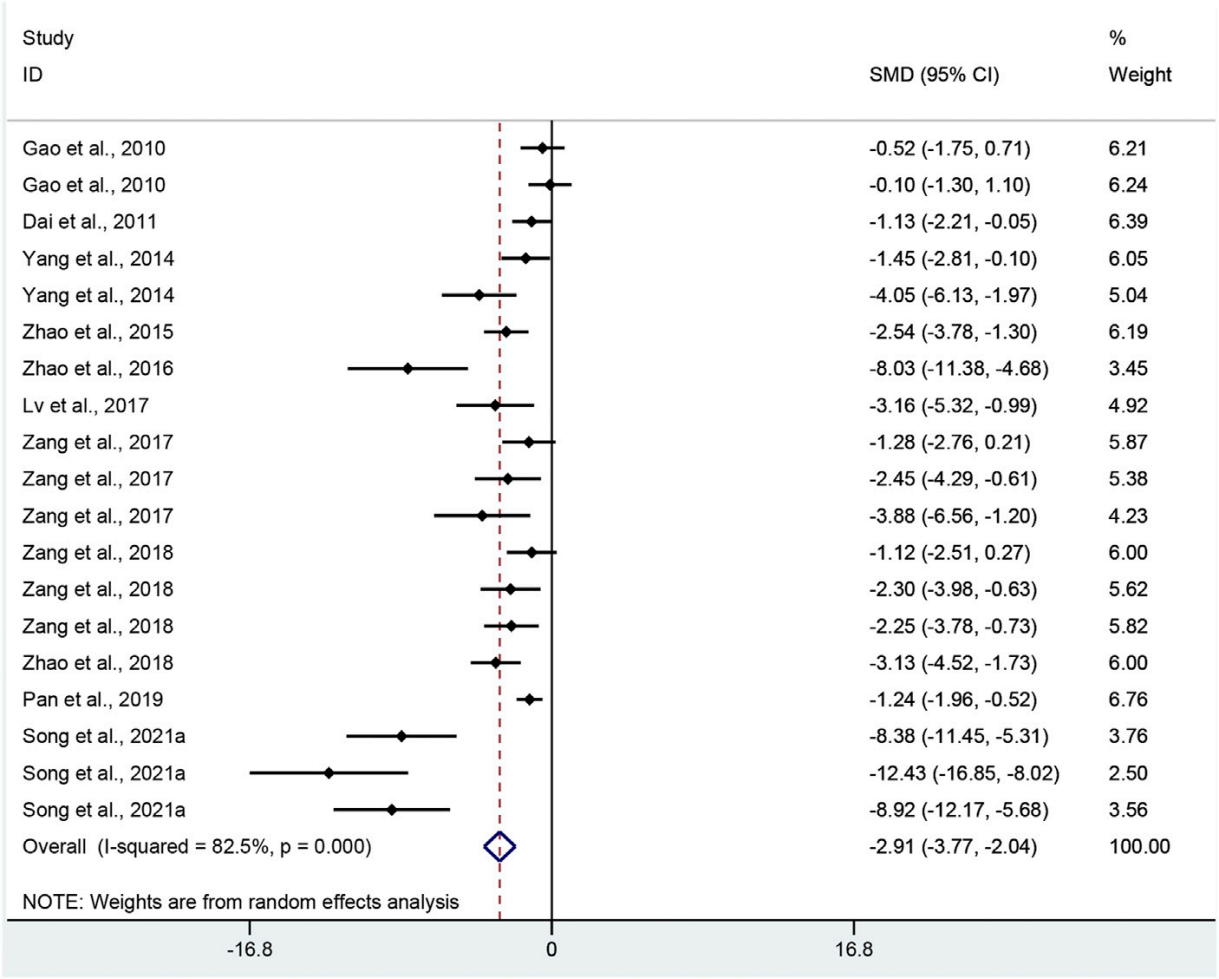
Pooled estimate of CHS with APS.

CHS: Nineteen pair-wise comparisons mentioned the impact of APS on CHS. This meta-analysis showed that APS could significantly decrease CHS scores compared with the control group (SMD = −2.91, 95% CI [−3.77, −2.04], *p =* 0.000; Heterogeneity: Chi^2^ = 103, *p* = 0.000; *I* ^2^ = 82.5% Figure 4).

**FIGURE 5:**
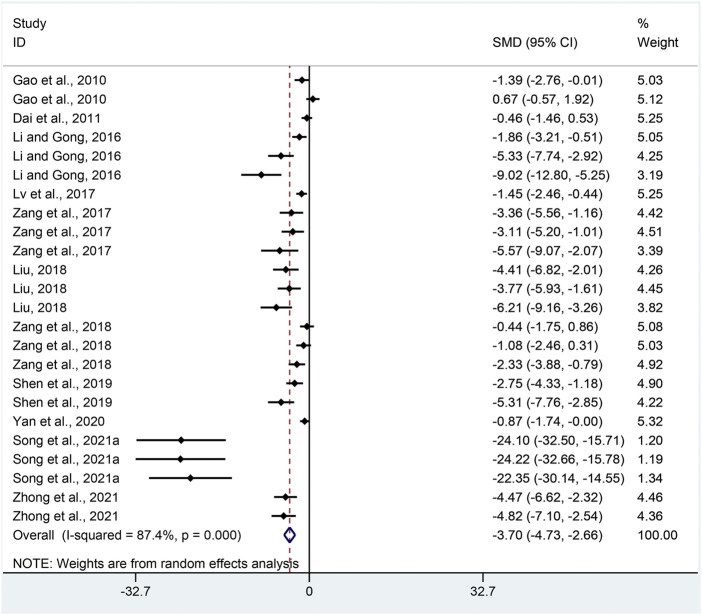
Pooled estimate of MPO with APS.

MPO: Twenty-four pair-wise comparisons mentioned the influence of APS on MPO. The pooled effect sizes indicated that APS could significantly decrease MPO activity compared with the control group (SMD = −3.70, 95% CI [−4.73, −2.66], *p* = 0.000; Heterogeneity: Chi^2^ = 181.98, *p* = 0.000; *I* ^2^ = 87.4% Figure 5).

**FIGURE 6:**
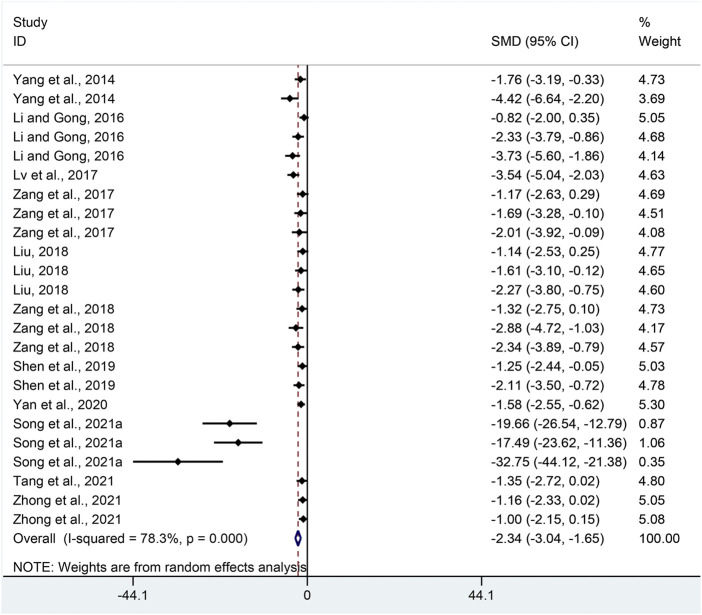
Pooled estimate of TNF-α with APS.

TNF-α: Combining effect sizes from twenty-four pair-wise comparisons, a significant reduction in TNF-α was recorded after APS treatment, compared to that in the control group (SMD = −2.34, 95% CI [−3.04, −1.65], *p* = 0.000; Heterogeneity: Chi^2^ = 106.08, *p* = 0.000; *I* ^2^ = 78.3% Figure 6).

**FIGURE 7:**
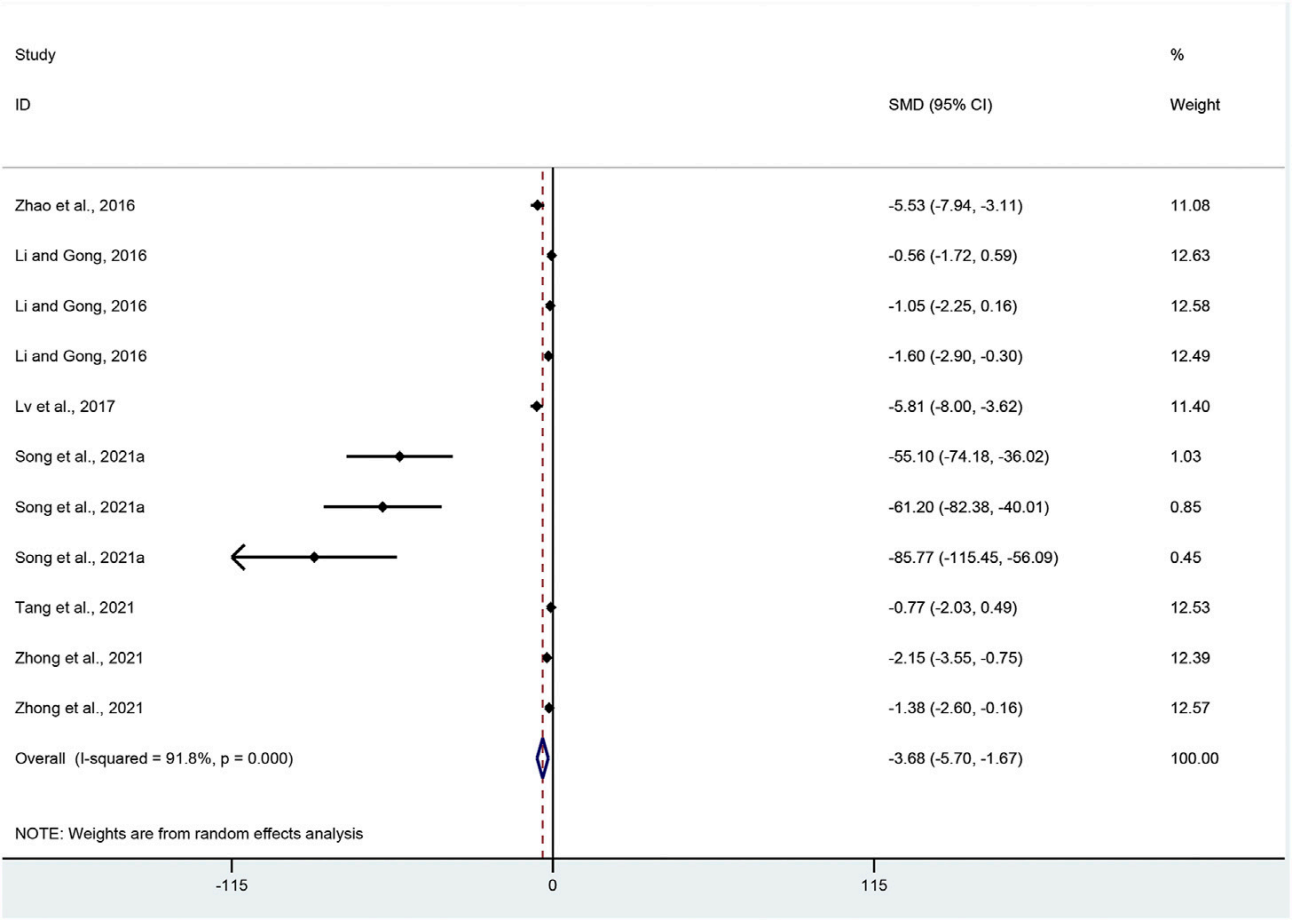
Pooled estimate of IL-6 with APS.

IL-6: With regard to the effect on IL-6 level, eleven pair-wise comparisons mentioned the influence of APS on this outcome. The pooled effect sizes showed that APS could significantly decrease IL-6 level compared with the control group (SMD = −3.68, 95% CI [−5.70, −1.67], *p* = 0.000; Heterogeneity: Chi^2^ = 121.98, *p* = 0.000; *I* ^2^ = 91.8% Figure 7).

**FIGURE 8:**
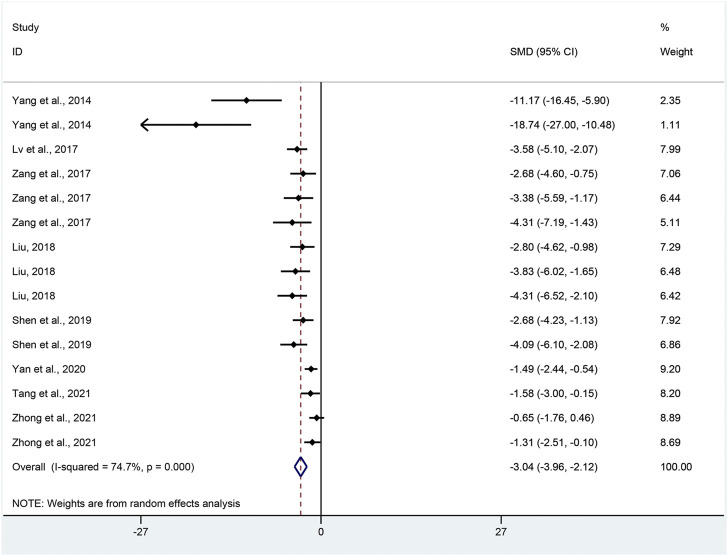
Pooled estimate of IL-1β with APS.

IL-1β: Effect sizes for IL-1β were pooled from a total of 15 pair-wise comparisons. There was a significant association of APS with IL-1β level (SMD = −3.04, 95% CI [−3.96, −2.12], *p* = 0.000; Heterogeneity: Chi^2^ = 55.28, *p* = 0.000; *I* ^2^ = 74.7% Figure 8).

**FIGURE 9:**
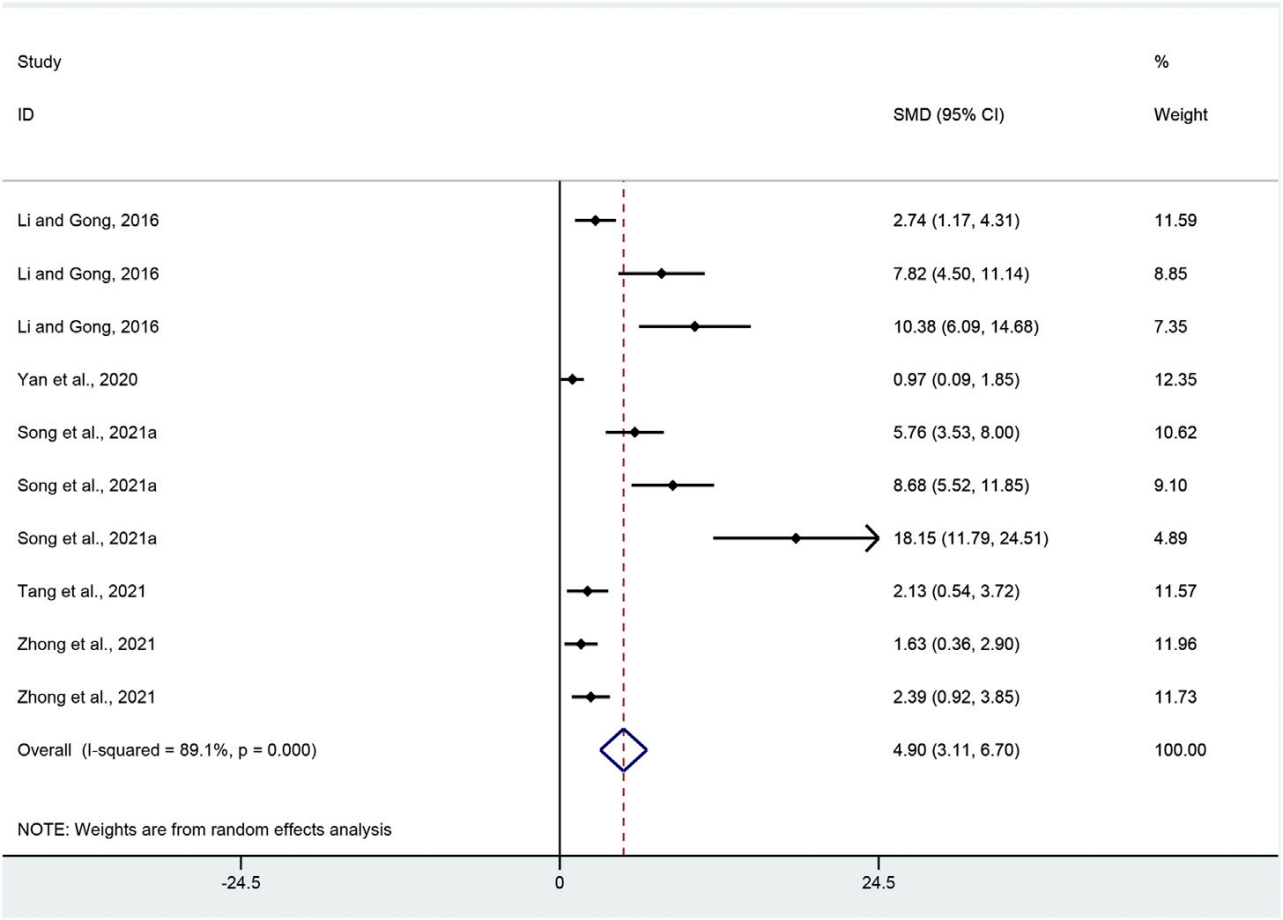
Pooled estimate of SOD with APS.

SOD: Ten pair-wise comparisons reported the impact of APS on SOD. The pooled effect sizes showed that APS could significantly increase SOD level compared with control group (SMD = 4.90, 95% CI [3.11, 6.70], *p* = 0.000; Heterogeneity: Chi^2^ = 82.62, *p* = 0.000; *I* ^2^ = 89.1% Figure 9).

**FIGURE 10:**
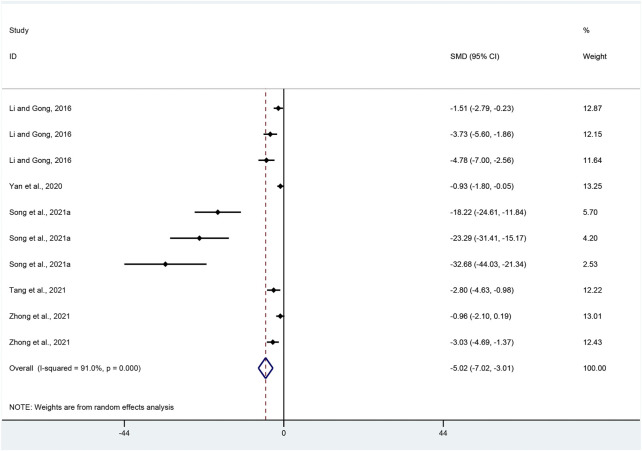
Pooled estimate of MDA with APS.

MDA: Effect sizes for MDA were pooled from a total of ten pair-wise comparisons. There was a significant association of APS with MDA level (SMD = −5.02, 95% CI [−7.02, −3.01], *p* = 0.000; Heterogeneity: Chi^2^ = 100.39, *p* = 0.000; *I* ^2^ = 91.0% Figure 10).

### Subgroup analysis

DAI: Subgroup analysis was conducted according to UC models, dosage, intervention duration and species. More beneficial effects were recorded when studies employed DSS-induced UC (*p* = 0.000), medium dosage (*p* = 0.000), intervention duration of ≤ 7 days (*p* = 0.001) and mice (*p* = 0.000) (Supplementary Table S1).

CMDI: The included studies were stratified based on variables including UC models, dosage and intervention duration. Better therapeutic effects of APS administration were observed when studies used DNCB-induced UC models (*p* = 0.000), medium dose (*p* = 0.006) and intervention duration of ≤ 7 days (*p* = 0.000) (Supplementary Table S1).

CHS: Subgroup analysis was conducted according to UC models, dosage, intervention duration and species. More beneficial effects were observed when studies employed DSS-induced UC (*p* = 0.000), medium dosage (*p* = 0.000), intervention duration of > 7 days (*p* = 0.000) and mice (*p* = 0.000) (Supplementary Table S1).

MPO: Subgroup analysis was conducted based on UC models, intervention duration, dosage and species. More beneficial effects were demonstrated when studies applied DSS-induced UC (*p* = 0.000), intervention duration of > 7 days (*p =* 0.000) and high dosage (*p* = 0.000), as well as studies that employed mice (*p* = 0.000) (Supplementary Table S1).

TNF-α: Subgroup analysis was conducted based on UC models, intervention duration, dosage and species. More beneficial effects were demonstrated when studies applied DSS-induced UC (*p* = 0.000), intervention duration of > 7 days (*p =* 0.000) and medium dosage (*p* = 0.000), as well as studies that employed mice (*p* = 0.000) (Supplementary Table S1).

IL-6: The included studies were stratified based on variables including UC models, dosage, intervention duration and species. More beneficial effects were observed when studies used DSS-induced UC (*p* = 0.000), medium dose (*p* = 0.018), duration of > 7 days and mice (*p* = 0.000) (Supplementary Table S1).

IL-1β: Subgroup analysis was performed in terms of UC models, duration, dosage and species. More beneficial effects were recorded when studies employed TNBS-induced UC (*p* = 0.000), intervention duration of ≤ 7 days (*p =* 0.001) and medium dosage (*p* = 0.000), as well as studies that employed rat (*p* = 0.000) (Supplementary Table S1).

SOD: Subgroup analysis was performed in terms of UC models, duration, dosage and species. More beneficial effects were recorded when studies employed DSS-induced UC (*p* = 0.000), intervention duration of > 7 days (*p =* 0.000) and high dosage (*p* = 0.033), as well as studies that employed mice (*p* = 0.000) (Supplementary Table S1).

MDA: Subgroup analysis was conducted based on UC models, intervention duration, dosage and species. More beneficial effects were demonstrated when studies applied DSS-induced UC (*p* = 0.000), intervention duration of > 7 days (*p =* 0.000) and high dosage (*p* = 0.006), as well as studies that employed mice (*p* = 0.000) (Supplementary Table S1).

### Sensitivity analysis

With regard to all outcome measures, sensitivity analysis was performed by removing a single study at each stage and the pooled results suggested that no individual study significantly influenced the overall effect sizes (Supplementary Figures S1–S9).

### Publication bias

DAI: Visual inspection of funnel plots showed asymmetry for the effect of APS on DAI (Supplementary Figure S10), and the result of Egger’s test was statistically significant (intercept: −4.74, 95% CI [−5.78, −3.71]; *p* = 0.000).

CMDI: Visual inspection of funnel plots indicated asymmetry for the effect of APS on CMDI (Supplementary Figure S11), and the result of Egger’s test was statistically significant (intercept: −3.08, 95% CI [−5.11, −1.05]; *p* = 0.005).

CHS: Funnel plots indicated asymmetry for the effect of APS on CHS (Supplementary Figure S12), while the result of Egger’s test was statistically significant (intercept: −4.91, 95% CI [−6.55, −3.26]; *p* = 0.000).

MPO: Funnel plots suggested asymmetry for the influence of APS on MPO (Supplementary Figure S13), while the result of Egger’s test was statistically significant (intercept: −5.67, 95% CI [−6.62, −4.73]; *p* = 0.000).

TNF-α: Funnel plots suggested asymmetry for the effect of APS on TNF-α (Supplementary Figure S14), and the result of Egger’s test was statistically significant (intercept: −5.76, 95% CI [−6.82, −4.70]; *p* = 0.000).

IL-6: Funnel plots indicated asymmetry for the effect of APS on IL-6 (Supplementary Figure S15), while the result of Egger’s test was statistically significant (intercept: −6.21, 95% CI [−7.09, −5.34]; *p* = 0.000).

IL-1β: Funnel plots indicated asymmetry for the effect of APS on IL-1β (Supplementary Figure S16), while the result of Egger’s test was statistically significant (intercept: −4.45, 95% CI [−5.55, −3.36]; *p* = 0.000).

SOD: Funnel plots showed asymmetry for the effect of APS on SOD (Supplementary Figure S17), while the result of Egger’s test was statistically significant (intercept: 6.00, 95% CI [5.12, 6.88]; *p* = 0.000).

MDA: Funnel plots showed asymmetry for the effect of APS on MDA (Supplementary Figure S18), while the result of Egger’s test was statistically significant (intercept: −5.98, 95% CI [−6.68, −5.28]; *p* = 0.000).

## Discussion

### Efficacy of astragalus polysaccharide

The present systematic review and meta-analysis primarily intended to assess the anti-inflammatory and antioxidant activities of APS when used in the treatment of UC. The results showed that APS was significantly associated with a lower level of TNF-α, IL-6, IL-1β, MPO, and MDA and a higher level of SOD. In addition, sensitivity analysis that eliminated a single study at each stage did not change these results. On the basis of above findings, this meta-analysis demonstrated that APS could confer protection against experimental UC by inhibiting inflammation and decreasing oxidative stress.

### Implication for further studies

APS is a water-soluble component with greater polarity in *Astragalus membranaceus*. Currently, the extraction methods of APS consist of enzymatic hydrolysis, ultrasonic extraction and water extraction. However, water extraction is more common (Rui et al., 2019). After the extraction, APS should be further separated and purified. The methods of separation and purification consist of graded alcohol precipitation, gel column chromatography, macroporous resin adsorption and quaternary ammonium salt complex method (Liu et al., 2021). Because of the complicated extraction and purification process, the molecular weight distribution of the extracted APS is uneven. A recent work obtained two different molecular weight APS through extraction and purification: APS Ⅰ (molecular weight: 10.6 kDa) and APS Ⅱ (molecular weight: 2,470 kDa). APS Ⅰ is composed of mannose, rhamnose, glucuronic acid, galacturonic acid, glucose and galactose, and APS Ⅱ consists of mannose, rhamnose, glucuronic acid, galacturonic acid, glucose, galactose and xylose (Tang et al., 2014). Another study showed that APS contains APS Ⅰ (more than 2000 kDa) and APS II (10 kDa), and both are composed of rhamnose, glucose, galactose, arabinose and galacturonic acid. However, the proportion of each monosaccharide in APS I and APS II is different (Fan et al., 2021). Due to its broad pharmacological effects, APS has received high attention in recent years.

DAI, CMDI, and CHS scores can appropriately reflect the degree of colonic mucosal tissues damage. Several reports found that APS treatment could significantly reduce DAI, CMDI, and CHS scores in the animal models of UC (Yang et al., 2014; Pan et al., 2019). In this meta-analysis, compared to that in the control group, DAI, CMDI and CHS scores were all significantly decreased in the experimental group. These results indicated that APS could markedly mitigate colonic damage. Early studies also found that APS dose-dependently decreased the DAI, CMDI and CHS scores (Li and Gong, 2016; Zang et al., 2018; Song et al., 2021b). However, A study by Guo et al. (2013) reported that the effects of APS on DAI and CMDI scores did not appear dose-dependent. Therefore, conflicting results about the dose-response effects of APS in the treatment of UC still exist. In this meta-analysis, with regard to DAI, CMDI, and CHS, the greatest therapeutic effects were observed in medium-dose group rather than in low-dose group and high-dose group. In other words, APS efficacy will gradually increase with the rise of APS dose, and the optimal therapeutic effect will be recorded after APS dosage reach a specific threshold (medium dosage). The therapeutic effect of APS will decrease while APS dosage exceed this specific threshold. Based on this result, variability in APS dosage may have an impact on efficacy, and medium-dosage APS is more suitable for the management of UC. Furthermore, whether an excessive dose of APS will suppress its efficacy in the treatment of UC should be further studied. No study reported the time-response effects of APS when used in the treatment of UC. Our subgroup analysis suggested that intervention duration of APS may influence the DAI and CMDI, and greatest therapeutic effects were recorded when studies had intervention duration of ≤ 7 days. Nevertheless, with regard to CHS, more beneficial effects were observed when studies applied intervention duration of > 7 days. Thus, it is insufficient to determine the appropriate intervention duration according to above inconsistent findings. This result may be due to the small sample size in the subgroup analysis and the heterogeneity among animal studies. In the future studies, whether the therapeutic effects of APS are influenced by intervention duration still needs to be further investigated.

Cytokines are believed to be involved in the pathogenesis of UC, where they control multiple critical aspects of the inflammatory response (Neurath, 2014). Patients with UC exhibited higher levels of pro-inflammatory cytokines, including TNF-α, IL-1β, and IL-6, in colonic mucosal tissues (Ishiguro, 1999). TNF-α is considered to be the utmost powerful pro-inflammatory cytokines, and directly affects intestinal epithelial tissue. Furthermore, TNF-α activates the adaptive immune system of the intestine by recruiting and activating neutrophils and macrophages (Cho et al., 2011). IL-1β and IL-6 are also key mediators of the development of UC. Previous study found that APS was significantly associated with a lower level of TNF-α, IL-1β, and IL-6 (Song et al., 2021a). Results reported here were consistent with previous findings. A early study indicated that the therapeutic effects of APS on TNF-α level appear dose-dependent. Our meta-analysis showed that variability in APS dose could influence TNF-α level, and optimal efficacy of APS was found when studies applied medium-dose APS. As for IL-1β and IL-6, a great effect was also observed when studies had medium-dose APS. These results suggested the possibility of a significant association of APS dose with pro-inflammatory cytokines, and medium-dose APS could bring the greatest anti-inflammatory effects. For time-response effects, our subgroup analysis suggested that intervention duration of APS might have an influence on the level of TNF-α and IL-6, and greatest therapeutic effects were recorded when studies had intervention duration of > 7 days. However, with regard to IL-1β, more beneficial effects were observed when studies used intervention duration of ≤ 7 days. Small sample size may contribute to this inconsistent result. In the future studies, the time-response effects between APS and pro-inflammatory cytokines should still be clarified by increasing the sample size.

Another point that must be considered is anti-inflammatory cytokines, such as IL-10. The imbalance between pro-inflammatory cytokines and anti-inflammatory cytokines that occurs in UC prevents the improvement of inflammation and instead leads to disease perpetuation and tissue destruction (Neurath, 2014). However, few studies elucidated the efficacy of APS on anti-inflammatory cytokines. Hence, it is necessary to clarify the influence of APS on pro-inflammatory and anti-inflammatory cytokines in the future animal studies and clinical trials.

MPO is an enzyme with high content in neutrophils, and its activity changes represent the degree of neutrophil infiltration, so it has been used as a real measurable marker of inflammation in the colon (El-Shiekh et al., 2021). Increased MPO activity has been shown to hasten the progression of UC (Mandlik et al., 2021). Early reports showed that APS could significantly decrease MPO activity in experimental UC (Liu, 2018; Song et al., 2021a). Their findings were supported by our meta-analysis. In addition, A previous study reported that high-dose APS tended to have a better effect on MPO activity compared to low-dose APS. In the meta-analysis reported here, the highest effects were also recorded in high-dose group. For time-response effects, subgroup analysis indicated that intervention duration of APS could influence the MPO activity, more beneficial effects were observed when studies had intervention duration of > 7 days.

Oxidative stress is considered to be one of the etiologic factors involved in UC (Miyake et al., 2021). An imbalance between oxidative stress and antioxidant capacity, is believed to play an important role in modulating intestinal tissue damage (Zhao et al., 2021). SOD is an antioxidant enzyme found in mitochondria and the cytoplasm, which can keep the tissue’s redox balance in check (Mandlik et al., 2021). MDA is an unsaturated aldehyde produced by the oxidation of polyunsaturated fatty acids, and excessive MDA levels can cause structural modifications and immune responses (Mandlik et al., 2021). SOD and MDA are used *in-vivo* as a marker of oxidative stress. A previous study found that APS treatment could effectively mitigate colonic damage by reducing the MDA levels and recovering the SOD activity (Song et al., 2021a). Consistent with the previous findings, in the present study, reduced MDA levels and increased SOD levels were observed following APS treatment.

Inflammatory cytokines play an important role in the progression of UC. For the moment, the recognized pro-inflammatory cytokines mediating UC pathogenesis mainly include TNF-α, IL-1β, and IL-6. A clinical study found that the level of TNF-α was positively correlated with the degree of UC lesions (Wang et al., 2014). Another study also indicated that serum TNF-α levels in UC patients was significantly higher than those in the control group, and increased with the patient’s disease activity (Wu et al., 2017). TNF-α could activate nuclear factor-κB (NF-κB), which can promote the secretion of TNF-α and aggravate inflammatory response through the positive feedback (Berndt et al., 2007). Meanwhile, TNF-α can initiate cytotoxicity, apoptosis and acute phase reaction, and stimulate the secretion of IL-1β and IL-6, thus destroying the intestinal mucosal barrier (Sanchez-Munoz et al., 2008). IL-6 could activate signal transducer and activator of transcription-3 (STAT3) to induce anti-apoptotic factors BCL-XL and BCL-2, which can obstruct the apoptosis of T cells, and T cells gather in the inflammation areas, leading to the occurrence of UC (Jia and Guo, 2018). A Clinical study showed that serum IL-6 concentration in patients with UC was positively correlated with the severity of the UC (Zhang and Zhang, 2017). The primary function of IL-1β is to initiate and amplify inflammatory response. Normal intestinal mucosa produced very little mature IL-1β, while UC patients produced a large amount of mature IL-1β (Magyari et al., 2014). The increase of the above pro-inflammatory cytokines in UC is closely related to the adiponectin/TLR/NF-κB signaling pathway. A previous study suggested that APS can decrease TLR4 and NF-κBp65 levels and increase adiponectin levels in colonic tissues of mice with UC, thereby inhibiting the release of TNF-α and IL-6 (Song et al., 2021a). Another study indicated that the change of pro-inflammatory cytokines levels was correlated with nuclear factor of activated T cell-4 (NFATc4). After APS treatment, while TNF-α and IL-1β expression was down-regulated, NFATc4 mRNA and protein expression was further up-regulated (Yang et al., 2014). According to the discussion above, further studies are needed to clarify the exact role of adiponectin/TLR/NF-κB signaling pathway and NFATc4 in UC and how they interact with TNF-α, IL-1β, and IL-6. MPO is secreted by neutrophils, and its activity is an important indicator of neutrophils infiltration. Moreover, MPO can indirectly reflect the degree of intestinal inflammatory activity in UC patients (Xu et al., 2006). Oxidative stress is another important factor to promote the occurrence and development of UC. Free radicals are produced in the form of neutrophil “respiratory burst.” After a series of peroxidatic reaction, the final product of oxygenation is MDA, which can accelerate the activation of inflammatory cells by damaging biological macromolecules such as proteins and DNA, stimulate the overexpression of inflammatory cytokines (Li and Gong, 2016). SOD is an antioxidant enzyme that scavenge excess free radicals to protect cells. Therefore, inflammation and oxidative stress play an important role in the occurrence and development of UC. Meanwhile, these two aspects can influence each other, and are not completely separated. Thus, it is necessary to pay attention to the changes of inflammatory markers and oxidative stress markers at the same time when APS is applied to treat UC.

Every medical intervention is accompanied by adverse effects, thus attention should also be paid to whether APS had adverse effects. Early acute toxicity test showed that the acute, oral maximum-tolerated dosage (MTD) of APS to rats was more than 15.0 g/kg bw, which was equivalent to 300 times of the recommended value to human body. Furthermore, the 30-day feeding study indicated that all the doses of 1.25, 2.50, and 5.00 g/kg bw did not produce obvious adverse effects on the body weight, organ growth and blood biochemical indicators in rats. In addition, genotoxicity studies including mouse bone marrow cell micro-nucleus test, mouse sperm aberration test and Ames test were all negative (Zhaorigeritu et al., 2009). A previous study performed acute toxicity test and sub-chronic toxicity test on mice. In acute toxicity test, all mice survived healthily, and no damage was observed on the organs except the enlarged spleen. Moreover, no adverse effects were observed in sub-chronic toxicity test (Liu et al., 1996). According to these studies, APS had good safety and belonged to the category of non-toxic substances (Zhou et al., 2018). In this meta-analysis, no study mentioned the occurrence of adverse effects. The following two reasons could explain this result: the dosage and drug administration time of APS were within a appropriate range, which may not be sufficient to generate adverse effects. In addition, researcher did not report the occurrence of adverse events in the experiment. According to the aforementioned findings, it is important to focus on the following two aspects in the future studies: firstly, the adverse effects of APS are mainly confirmed in mice, rats and other animals, so caution is still needed in subsequent clinical trials. Secondly, some researchers did not report the occurrence of adverse events, which may lead others to believe that APS has no adverse effects. Therefore, whether there are adverse effects or not needs to be reported in the experiment.

Several limitations should be considered in this systematic review and meta-analysis. First, a number of studies did not report baseline characteristics between experimental group and control group. Second, Egger’s test and asymmetry of funnel plot showed that publication bias was existed, which could exaggerate the therapeutic effects of APS. Therefore, the positive finding of APS should be interpreted with caution. Third, some included studies were of poor quality. For example, some studies did not clarify randomization process in detail. Fourth, the accuracy of the dose-response effects and time-response effects might be affected because of the heterogeneity and small sample size.

## Conclusion

In this meta-analysis, APS treatment could significantly alleviate colonic damage by reducing the levels of MPO, TNF-α, IL-6, IL-1β, and MDA and recovering the SOD activity. These results demonstrated a protective role of APS in experimental UC and suggested that the anti-inflammatory and antioxidant activity were implicated in the underlying mechanisms. Therefore, APS may represent a promising candidate for the treatment of UC. However, due to potential publication bias, a cautious interpretation is needed.

## Data Availability

The original contributions presented in the study are included in the article/Supplementary Material, further inquiries can be directed to the corresponding authors.
