# Supplementary material for: Anti-inflammatory and antioxidant activity of astragalus polysaccharide in ulcerative colitis: A systematic review and meta-analysis of animal studies
Source: Front Pharmacol. 2022 Dec 2;13:1043236. doi: 10.3389/fphar.2022.1043236 (PMC9755193; doi:10.3389/fphar.2022.1043236)
Supplement: Supplementary file 2 [file Table1.DOCX]

**Table S1** Subgroup analysis for each outcome measures.

| **Variables** | **No. of Trials** | **SMD [95%CI]** | ***P* value** | ***I^2^*(%)** | ***P*-heterogeneity** | |
| --- | --- | --- | --- | --- | --- | --- |
| **DAI** |  |  |  |  |  | |
| UC models |  |  |  |  |  | |
| TNBS | 18 | -1.42 [-1.73, -1.10] | 0.000 | 8.9 | 0.350 | |
| DNCB | 4 | -1.69 [-2.41, -0.96] | 0.000 | 0.0 | 0.590 | |
| DSS | 11 | -6.12 [-8.38, -3.86] | 0.000 | 91.0 | 0.000 | |
| dosage |  |  |  |  | |  |
| low | 7 | -2.09 [-3.43, -0.76] | 0.002 | 80.5 | | 0.000 |
| medium | 8 | -3.07 [-4.43, -1.71] | 0.000 | 82.6 | | 0.000 |
| high | 15 | -1.66 [-2.34, -0.98] | 0.000 | 67.2 | | 0.000 |
| NR | 3 | -5.76 [-10.34, -1.18] | 0.014 | 91.3 | | 0.000 |
| duration |  |  |  |  | |  |
| ≤ 7 days | 7 | -3.19 [-5.00, -1.39] | 0.001 | 86.0 | | 0.000 |
| > 7 days | 26 | -2.00 [-2.58, -1.43] | 0.000 | 74.2 | | 0.000 |
| species |  |  |  |  | |  |
| rat | 22 | -1.44 [-1.72, -1.17] | 0.000 | 0.3 | | 0.455 |
| mice | 11 | -6.12 [-8.38, -3.86] | 0.000 | 91.0 | | 0.000 |
| **CMDI** |  |  |  |  | |  |
| UC models |  |  |  |  | |  |
| TNBS | 14 | -1.39 [-1.85, -0.93] | 0.000 | 51.4 | | 0.014 |
| DNCB | 4 | -1.75 [-2.48, -1.02] | 0.000 | 0.0 | | 0.917 |
| dosage |  |  |  |  | |  |
| low | 3 | -0.93 [-1.63, -0.23] | 0.009 | 0.0 | | 0.449 |
| medium | 3 | -1.87 [-3.19, -0.55] | 0.006 | 69.6 | | 0.037 |
| high | 11 | -1.58 [-2.13, -1.03] | 0.000 | 45.0 | | 0.052 |
| NR | 1 | -1.14 [-2.04, -0.24] | 0.013 | － | | － |
| duration |  |  |  |  | |  |
| ≤ 7 days | 4 | -2.60 [-4.05, -1.16] | 0.000 | 72.0 | | 0.013 |
| > 7 days | 14 | -1.21 [-1.52, -0.90] | 0.000 | 0.0 | | 0.460 |
| **CHS** |  |  |  |  | |  |
| UC models |  |  |  |  | |  |
| TNBS | 15 | -1.96 [-2.61, -1.32] | 0.000 | 67.1 | | 0.000 |
| DSS | 4 | -7.96 [-11.93, -4.00] | 0.000 | 84.7 | | 0.000 |
| dosage |  |  |  |  |  | |
| low | 4 | -2.61 [-4.71, -0.51] | 0.015 | 84.4 | 0.000 | |
| medium | 7 | -3.34 [-4.82, -1.87] | 0.000 | 81.8 | 0.000 | |
| high | 8 | -2.86 [-4.36, -1.36] | 0.000 | 85.7 | 0.000 | |
| duration |  |  |  |  |  | |
| ≤ 7 days | 9 | -2.70 [-3.57, -1.83] | 0.000 | 59.9 | 0.011 | |
| > 7 days | 10 | -3.19 [-4.61, -1.76] | 0.000 | 88.1 | 0.000 | |
| species |  |  |  |  |  | |
| rat | 14 | -1.86 [-2.52, -1.21] | 0.000 | 65.7 | 0.000 | |
| mice | 5 | -6.81 [-9.98, -3.64] | 0.000 | 87.0 | 0.000 | |
| **MPO** |  |  |  |  |  | |
| UC models |  |  |  |  |  | |
| TNBS | 18 | -2.68 [-3.58, -1.79] | 0.000 | 80.6 | 0.000 | |
| DSS | 6 | -11.42 [-16.30, -6.54] | 0.000 | 94.2 | 0.000 | |
| dosage |  |  |  |  |  | |
| low | 6 | -3.62 [-5.83, -1.42] | 0.001 | 86.9 | 0.000 | |
| medium | 6 | -4.00 [-6.26, -1.74] | 0.001 | 87.5 | 0.000 | |
| high | 9 | -4.25 [-6.34, -2.15] | 0.000 | 90.5 | 0.000 | |
| NR | 3 | -3.25 [-6.18, -0.31] | 0.030 | 88.3 | 0.000 | |
| duration |  |  |  |  |  | |
| ≤ 7 days | 6 | -2.18[-3.38, -0.99] | 0.000 | 73.4 | 0.002 | |
| > 7 days | 18 | -4.57 [-6.00, -3.14] | 0.000 | 89.6 | 0.000 | |
| species |  |  |  |  |  | |
| rat | 18 | -2.68 [-3.58, -1.79] | 0.000 | 80.6 | 0.000 | |
| mice | 6 | -11.42 [-16.30, -6.54] | 0.000 | 94.2 | 0.000 | |
| **SOD** |  |  |  |  |  | |
| UC models |  |  |  |  |  | |
| TNBS | 4 | 4.89 [1.64, 8.14] | 0.003 | 90.8 | 0.000 | |
| DSS | 6 | 5.16 [2.66, 7.67] | 0.000 | 89.2 | 0.000 | |
| dosage |  |  |  |  |  | |
| low | 2 | 4.14 [1.19, 7.10] | 0.006 | 78.7 | 0.030 | |
| medium | 2 | 8.27 [5.98, 10.57] | 0.000 | 0.0 | 0.713 | |
| high | 3 | 9.81 [0.80, 18.83] | 0.033 | 93.9 | 0.000 | |
| NR | 3 | 1.49 [0.70, 2.29] | 0.000 | 28.3 | 0.248 | |
| duration |  |  |  |  |  | |
| ≤ 7 days | 3 | 2.00 [1.18, 2.83] | 0.000 | 0.0 | 0.736 | |
| > 7 days | 7 | 7.01 [3.89, 10.12] | 0.000 | 92.5 | 0.000 | |
| species |  |  |  |  |  | |
| rat | 4 | 4.89 [1.64, 8.14] | 0.003 | 90.8 | 0.000 | |
| mice | 6 | 5.16 [2.66, 7.67] | 0.000 | 89.2 | 0.000 | |
| **MDA** |  |  |  |  |  | |
| UC models |  |  |  |  |  | |
| TNBS | 4 | -2.51 [-4.13, -0.88] | 0.002 | 80.0 | 0.002 | |
| DSS | 6 | -9.86 [-14.17, -5.56] | 0.000 | 94.0 | 0.000 | |
| dosage |  |  |  |  |  | |
| low | 2 | -9.56 [-25.93, 6.81] | 0.252 | 96.1 | 0.000 | |
| medium | 2 | -13.10 [-32.24, 6.05] | 0.180 | 95.3 | 0.000 | |
| high | 3 | -9.07 [-15.50, -2.65] | 0.006 | 92.5 | 0.000 | |
| NR | 3 | -1.46 [-2.56, -0.35] | 0.010 | 61.6 | 0.074 | |
| duration |  |  |  |  |  | |
| ≤ 7 days | 3 | -2.14 [-3.58, -0.70] | 0.004 | 62.6 | 0.069 | |
| > 7 days | 7 | -8.08 [-11.40, -4.75] | 0.000 | 93.7 | 0.000 | |
| species |  |  |  |  |  | |
| rat | 4 | -2.51 [-4.13, -0.88] | 0.002 | 80.0 | 0.002 | |
| mice | 6 | -9.86 [-14.17, -5.56] | 0.000 | 94.0 | 0.000 | |
| **TNF-α** |  |  |  |  |  | |
| UC models |  |  |  |  |  | |
| TNBS | 17 | -1.82 [-2.19, -1.44] | 0.000 | 11.5 | 0.320 | |
| DSS | 7 | -6.60 [-9.52, -3.69] | 0.000 | 93.2 | 0.000 | |
| dosage |  |  |  |  |  | |
| low | 7 | -1.77 [-3.03, -0.51] | 0.006 | 78.9 | 0.000 | |
| medium | 7 | -3.42 [-4.99, -1.86] | 0.000 | 79.8 | 0.000 | |
| high | 7 | -2.82 [-4.40, -1.24] | 0.000 | 81.1 | 0.000 | |
| NR | 3 | -1.29 [-1.92, -0.66] | 0.000 | 0.0 | 0.723 | |
| duration |  |  |  |  |  | |
| ≤ 7 days | 9 | -2.01 [-2.70, -1.31] | 0.000 | 50.1 | 0.042 | |
| > 7 days | 15 | -2.78 [-3.89, -1.68] | 0.000 | 84.5 | 0.000 | |
| species |  |  |  |  |  | |
| rat | 17 | -1.82 [-2.19, -1.44] | 0.000 | 11.5 | 0.320 | |
| mice | 7 | -6.60 [-9.52, -3.69] | 0.000 | 93.2 | 0.000 | |
| **IL-6** |  |  |  |  |  | |
| UC models |  |  |  |  |  | |
| TNBS | 4 | -1.87 [-3.38, -0.36] | 0.015 | 78.1 | 0.003 | |
| DSS | 7 | -7.74 [-11.78, -3.71] | 0.000 | 94.4 | 0.000 | |
| dosage |  |  |  |  |  | |
| low | 2 | -26.96 [-80.38, 26.45] | 0.322 | 96.8 | 0.000 | |
| medium | 3 | -9.32 [-17.02, -1.63] | 0.018 | 95.4 | 0.000 | |
| high | 4 | -4.03 [-8.08, 0.02] | 0.051 | 93.0 | 0.000 | |
| NR | 2 | -1.71 [-2.63, -0.79] | 0.000 | 0.0 | 0.416 | |
| duration |  |  |  |  |  | |
| ≤ 7 days | 5 | -2.90 [-4.64, -1.15] | 0.001 | 83.6 | 0.000 | |
| > 7 days | 6 | -7.73 [-12.20, -3.25] | 0.001 | 94.7 | 0.000 | |
| species |  |  |  |  |  | |
| rat | 4 | -1.87 [-3.38, -0.36] | 0.015 | 78.1 | 0.003 | |
| mice | 7 | -7.74 [-11.78, -3.71] | 0.000 | 94.4 | 0.000 | |
| **IL-1β** |  |  |  |  |  | |
| UC models |  |  |  |  |  | |
| TNBS | 11 | -3.79 [-5.02, -2.57] | 0.000 | 72.8 | 0.000 | |
| DSS | 4 | -1.70 [-2.87, -0.54] | 0.004 | 68.7 | 0.022 | |
| dosage |  |  |  |  |  | |
| low | 4 | -3.57 [-5.51, -1.63] | 0.000 | 68.5 | 0.023 | |
| medium | 4 | -4.78 [-7.37, -2.19] | 0.000 | 76.5 | 0.005 | |
| high | 4 | -3.37 [-4.95, -1.78] | 0.000 | 57.7 | 0.069 | |
| NR | 3 | -1.18 [-1.80, -0.56] | 0.000 | 0.0 | 0.513 | |
| duration |  |  |  |  |  | |
| ≤ 7 days | 6 | -3.47 [-5.53, -1.41] | 0.001 | 87.0 | 0.000 | |
| > 7 days | 9 | -2.98 [-3.76, -2.21] | 0.000 | 36.9 | 0.123 | |
| species |  |  |  |  |  | |
| rat | 11 | -3.79 [-5.02, -2.57] | 0.000 | 72.8 | 0.000 | |
| mice | 4 | -1.70 [-2.87, -0.54] | 0.004 | 68.7 | 0.022 | |
