# Supplementary material for: Anti-inflammatory and antioxidant activity of astragalus polysaccharide in ulcerative colitis: A systematic review and meta-analysis of animal studies
Source: Front Pharmacol. 2022 Dec 2;13:1043236. doi: 10.3389/fphar.2022.1043236 (PMC9755193; doi:10.3389/fphar.2022.1043236)
Supplement: Supplementary file 3 [file DataSheet1.PDF]

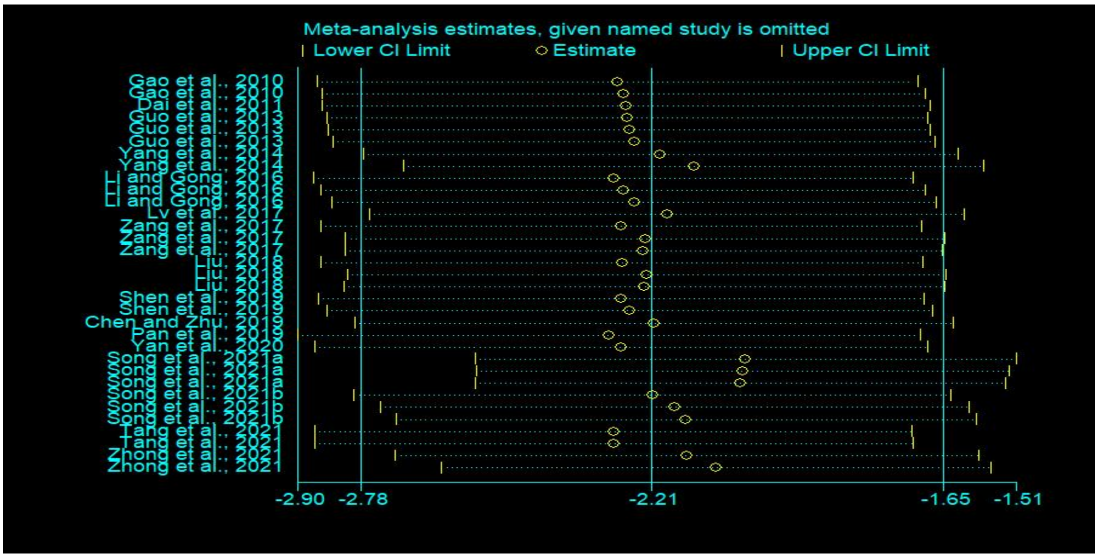

Figure S1. Results of sensitivity analysis according to DAI.

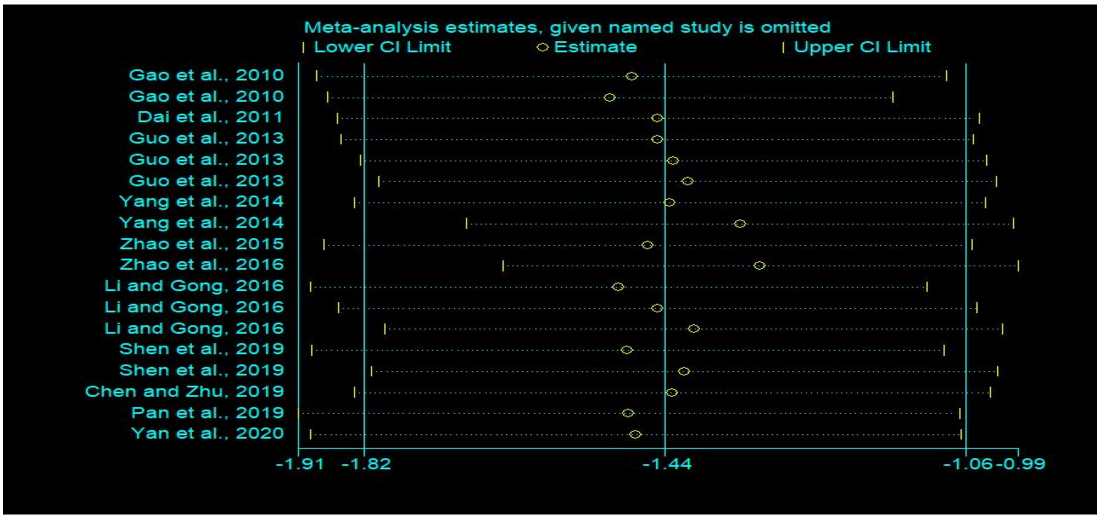

Figure S2. Results of sensitivity analysis according to CMDI.

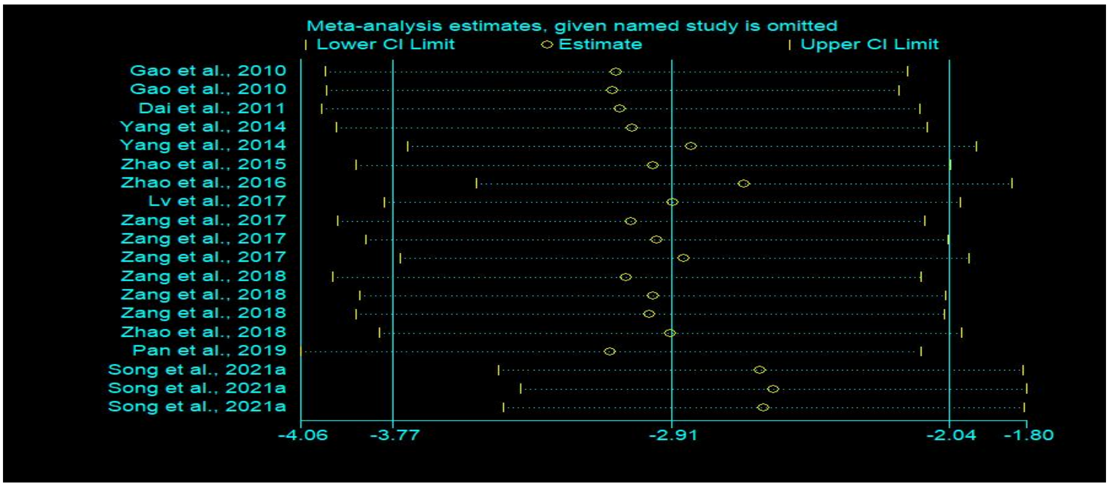

Figure S3. Results of sensitivity analysis according to CHS.

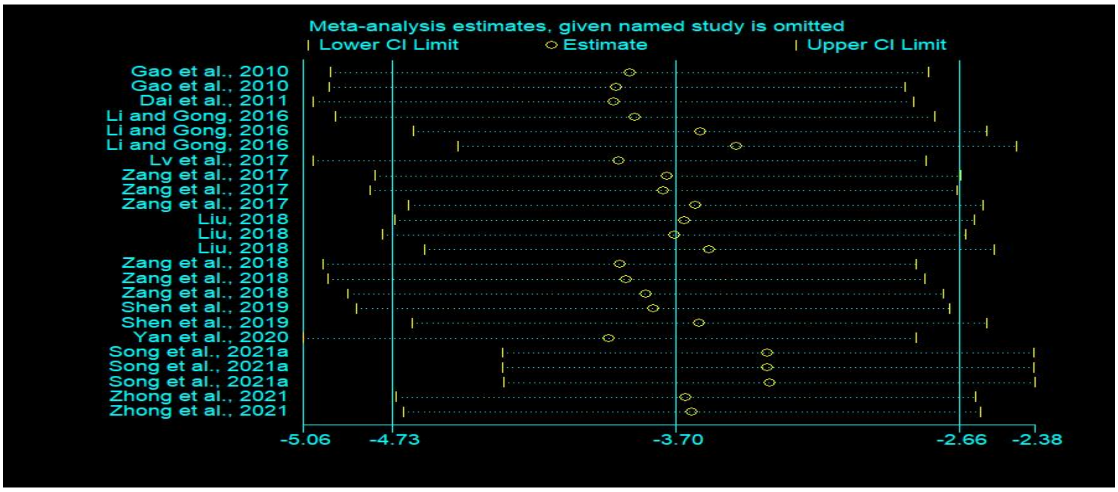

Figure S4. Results of sensitivity analysis according to MPO.

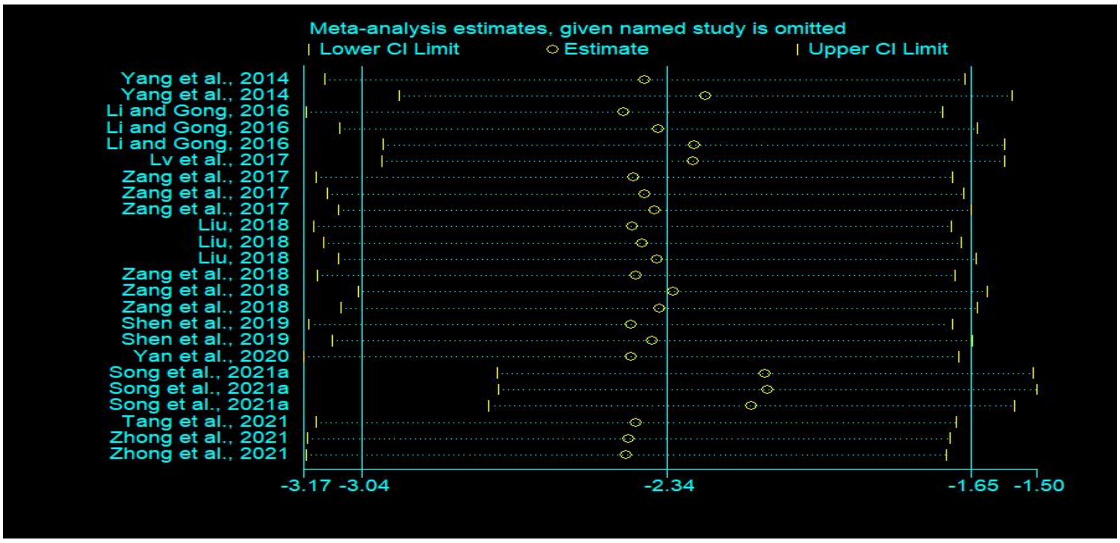

Figure S5. Results of sensitivity analysis according to TNF-α.

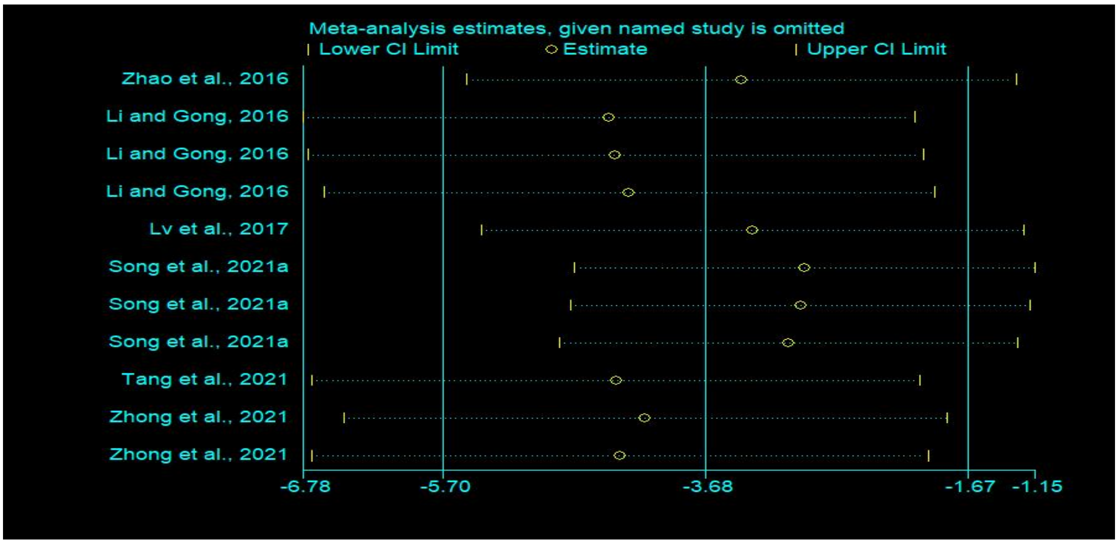

Figure S6. Results of sensitivity analysis according to IL-6.

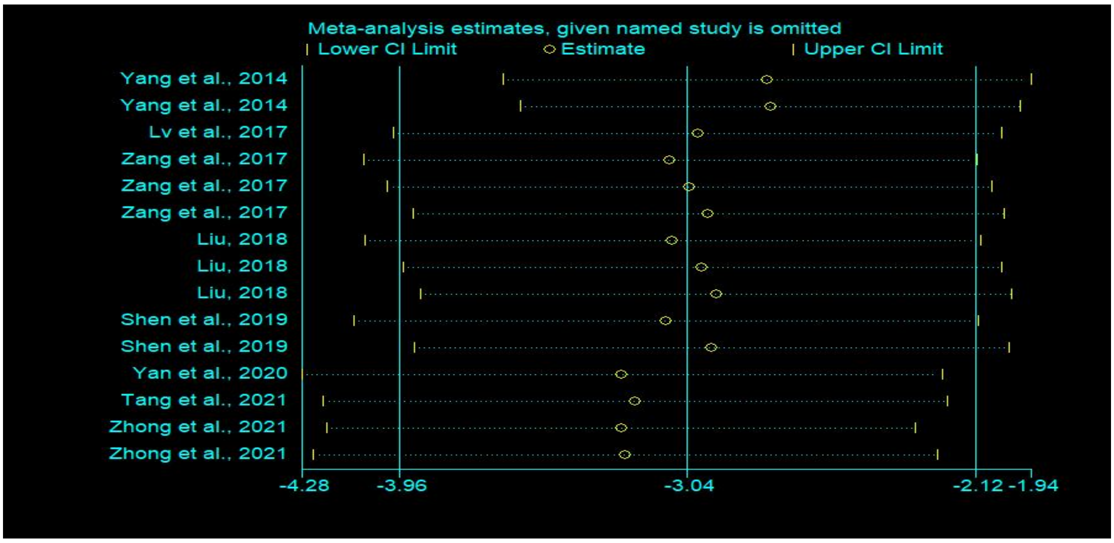

Figure S7. Results of sensitivity analysis according to IL-1β.

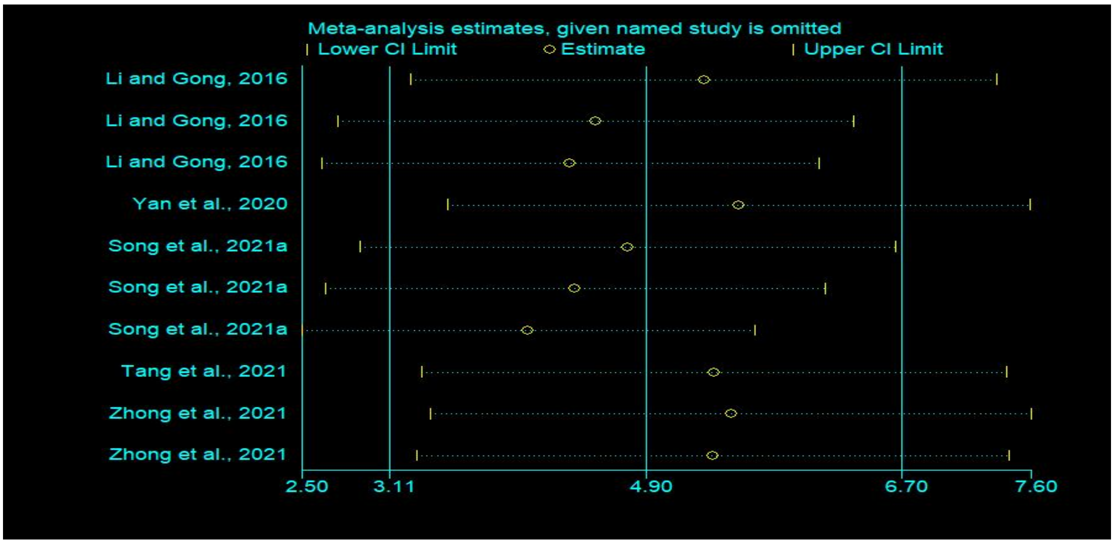

Figure S8. Results of sensitivity analysis according to SOD.

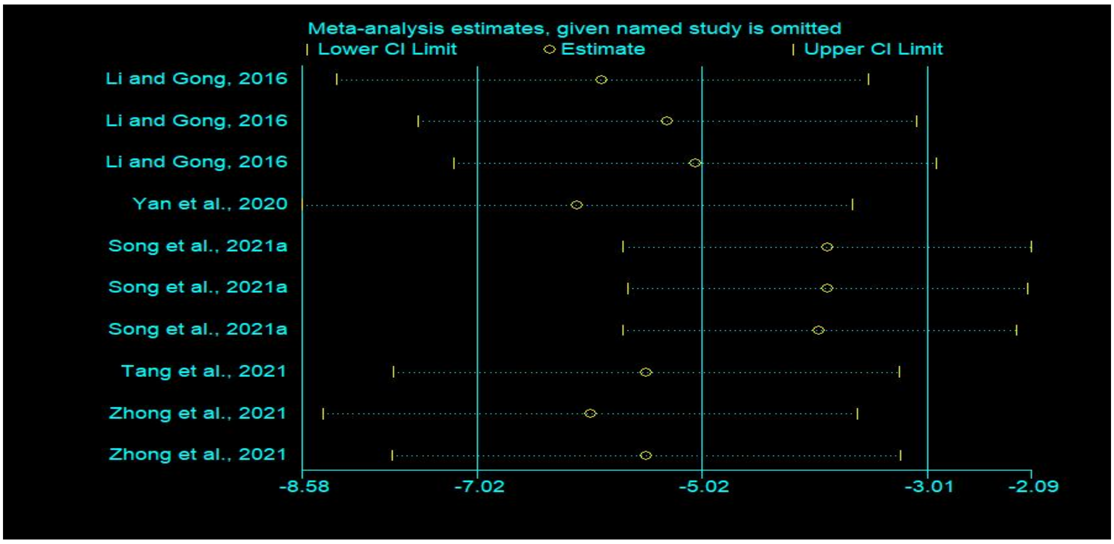

Figure S9. Results of sensitivity analysis according to MDA.

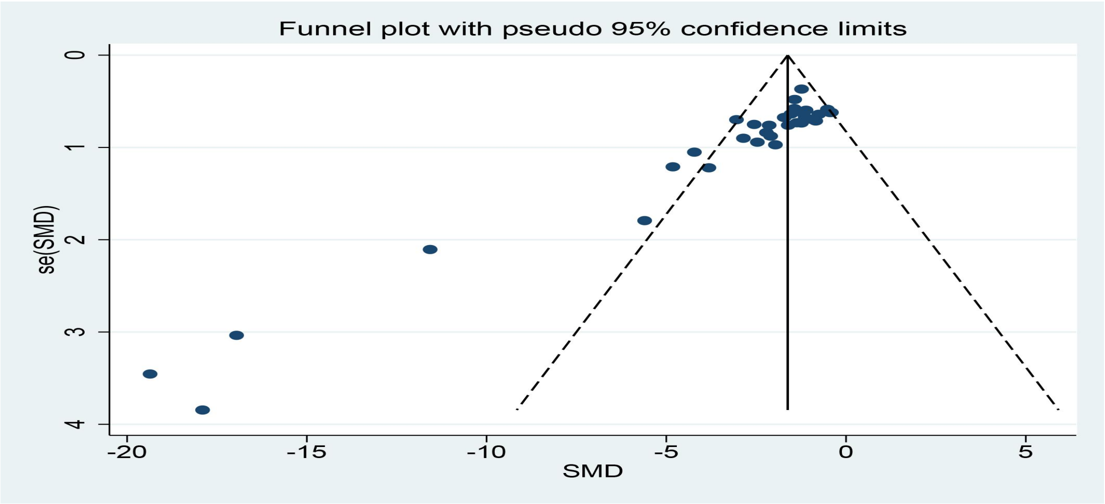

Figure S10. Funnel plot for efficacy of APS on DAI.

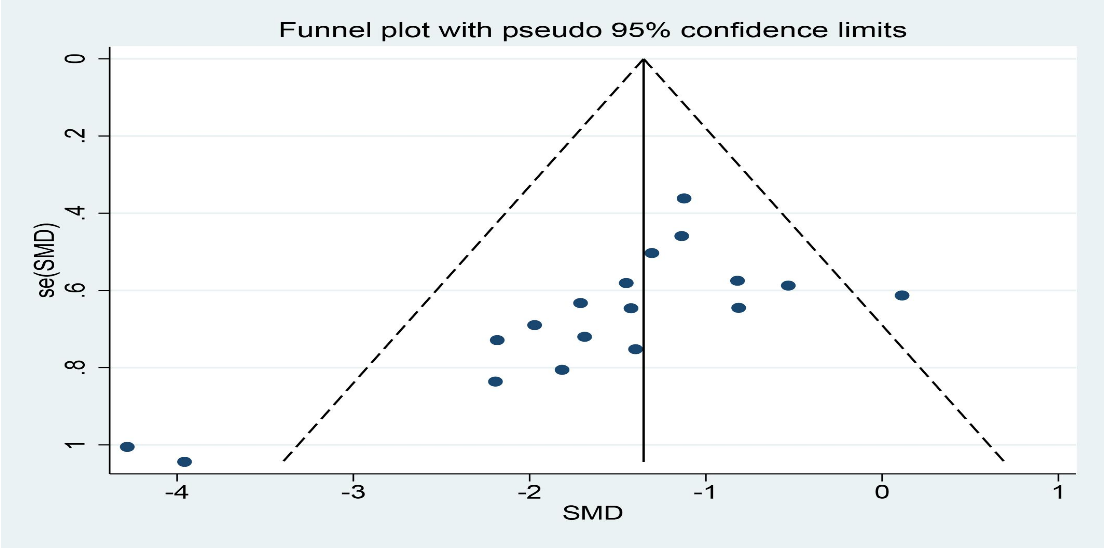

Figure S11. Funnel plot for efficacy of APS on CMDI.

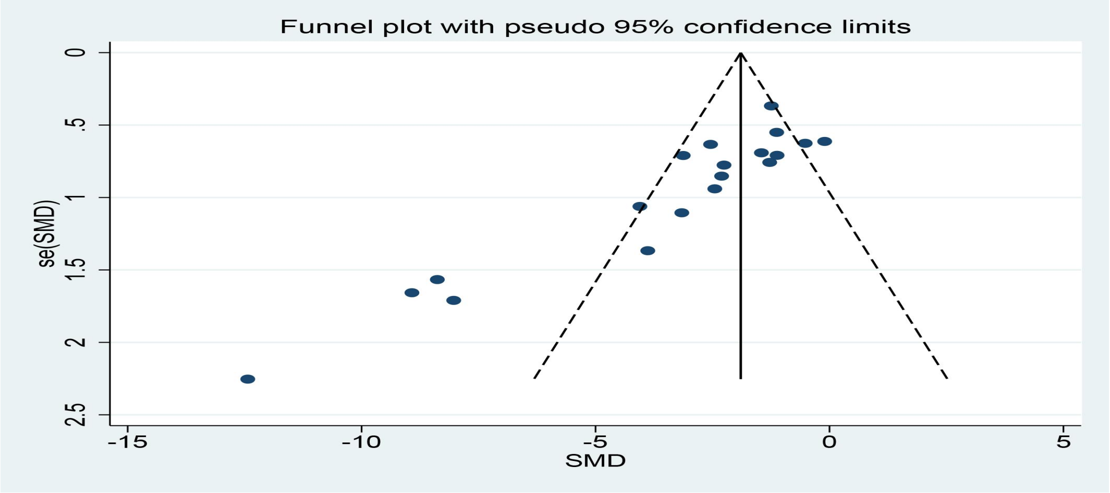

Figure S12. Funnel plot for efficacy of APS on CHS.

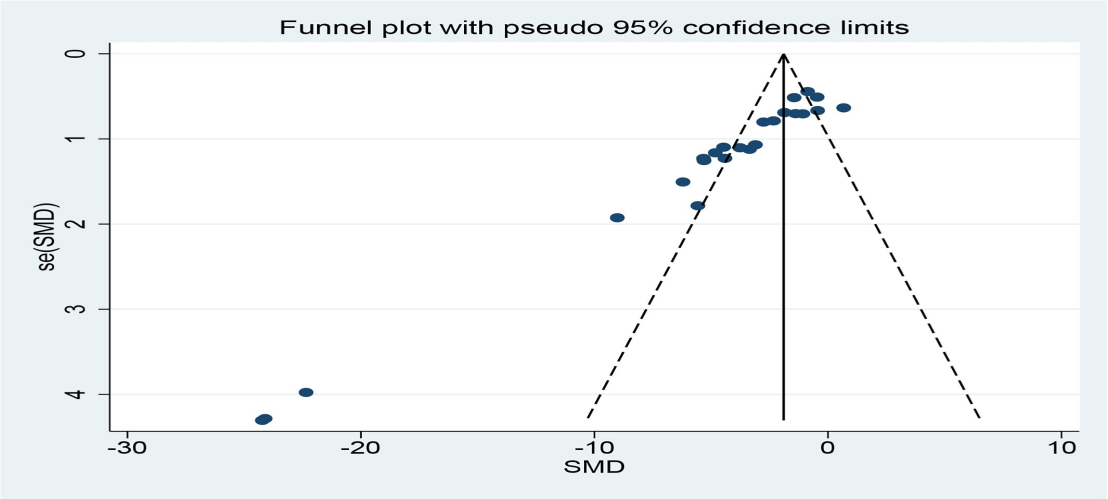

Figure S13. Funnel plot for efficacy of APS on MPO.

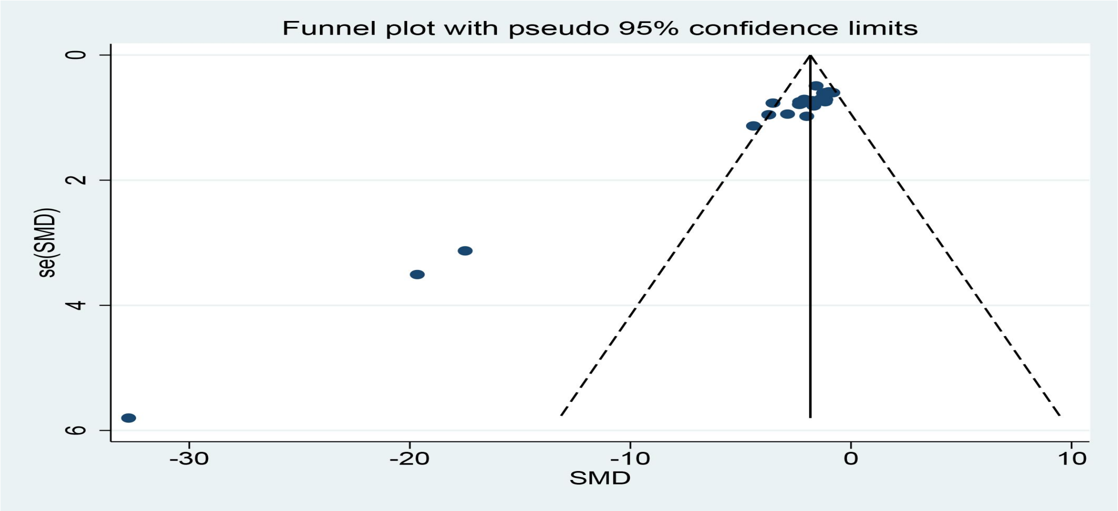

Figure S14. Funnel plot for efficacy of APS on TNF-α.

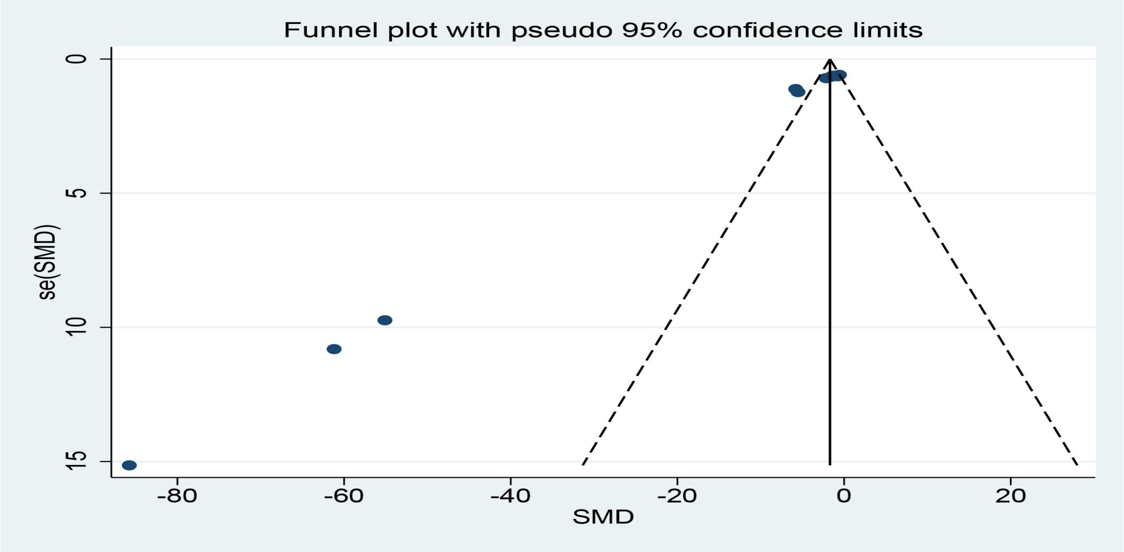

Figure S15. Funnel plot for efficacy of APS on IL-6.

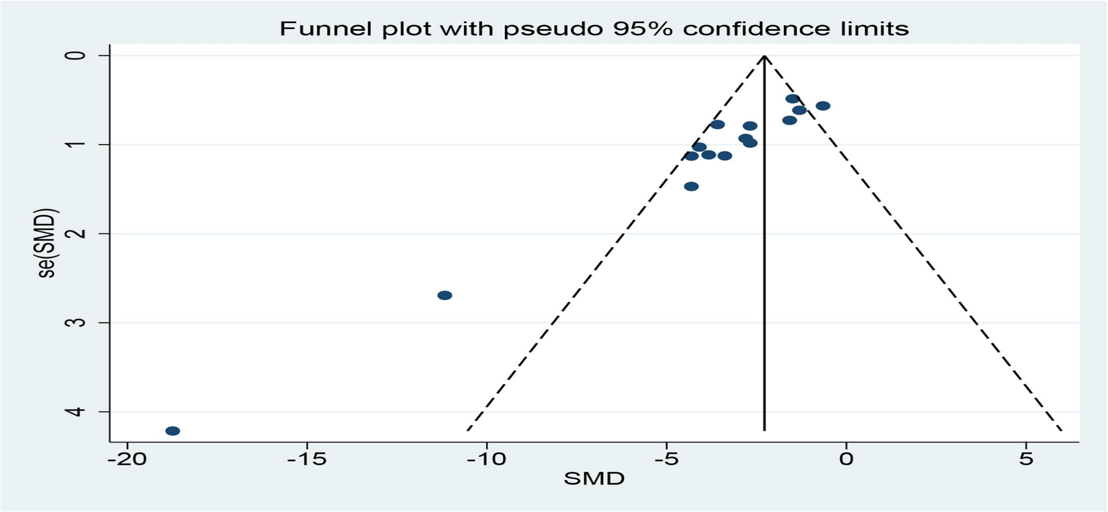

Figure S16. Funnel plot for efficacy of APS on IL-1β.

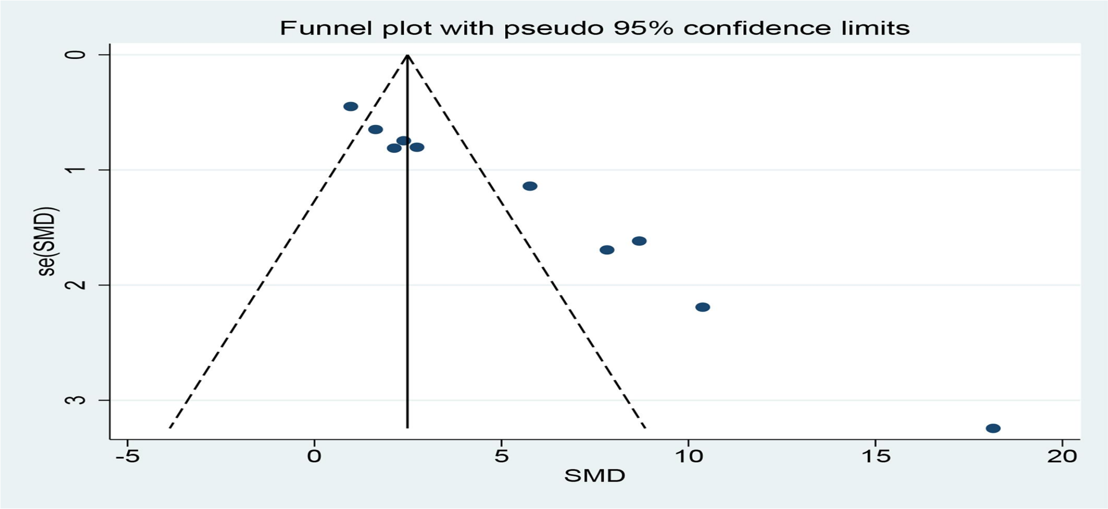

Figure S17. Funnel plot for efficacy of APS on SOD.

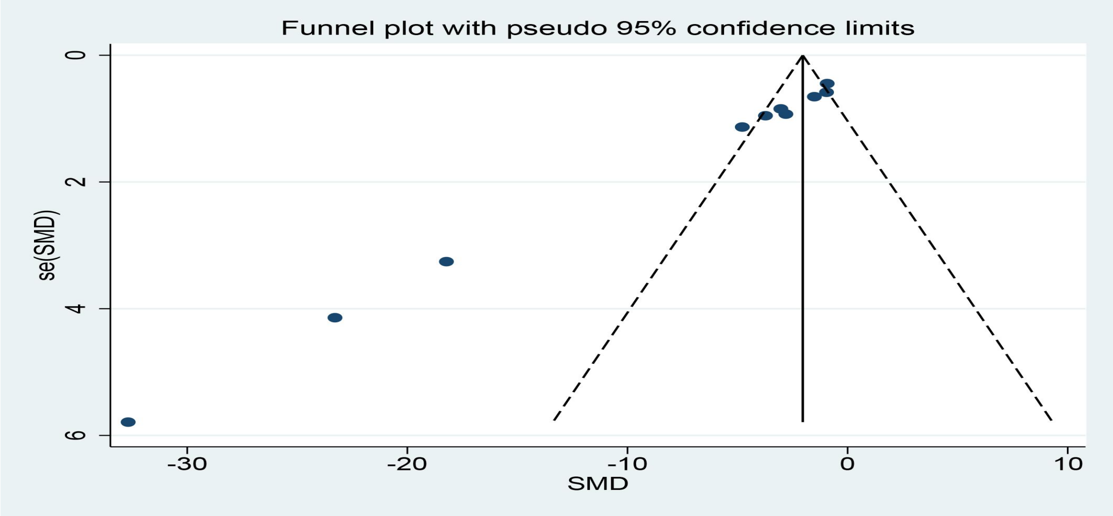

Figure S18. Funnel plot for efficacy of APS on MDA.
